# Knowledge transfer via classification rules using functional mapping for integrative modeling of gene expression data

**DOI:** 10.1186/s12859-015-0643-8

**Published:** 2015-07-23

**Authors:** Henry A. Ogoe, Shyam Visweswaran, Xinghua Lu, Vanathi Gopalakrishnan

**Affiliations:** 1 Department of Biomedical Informatics, University of Pittsburgh, Pittsburgh, USA; 2 Intelligent Systems Program, University of Pittsburgh, Pittsburgh, USA; 3 Department of Computational & Systems Biology, University of Pittsburgh, Pittsburgh, USA

**Keywords:** Transfer learning, Knowledge transfer, Functional modules, Functional mapping, Classification rules, Integrative modeling, Gene expression, Biomarker discovery

## Abstract

**Background:**

Most ‘transcriptomic’ data from microarrays are generated from small sample sizes compared to the large number of measured biomarkers, making it very difficult to build accurate and generalizable disease state classification models. Integrating information from different, but related, ‘transcriptomic’ data may help build better classification models. However, most proposed methods for integrative analysis of ‘transcriptomic’ data cannot incorporate domain knowledge, which can improve model performance. To this end, we have developed a methodology that leverages transfer rule learning and functional modules, which we call TRL-FM, to capture and abstract domain knowledge in the form of classification rules to facilitate integrative modeling of multiple gene expression data. TRL-FM is an extension of the transfer rule learner (TRL) that we developed previously. The goal of this study was to test our hypothesis that “an integrative model obtained via the TRL-FM approach outperforms traditional models based on single gene expression data sources”.

**Results:**

To evaluate the feasibility of the TRL-FM framework, we compared the area under the ROC curve (AUC) of models developed with TRL-FM and other traditional methods, using 21 microarray datasets generated from three studies on brain cancer, prostate cancer, and lung disease, respectively. The results show that TRL-FM statistically significantly outperforms TRL as well as traditional models based on single source data. In addition, TRL-FM performed better than other integrative models driven by meta-analysis and cross-platform data merging.

**Conclusions:**

The capability of utilizing transferred abstract knowledge derived from source data using feature mapping enables the TRL-FM framework to mimic the human process of learning and adaptation when performing related tasks. The novel TRL-FM methodology for integrative modeling for multiple ‘transcriptomic’ datasets is able to intelligently incorporate domain knowledge that traditional methods might disregard, to boost predictive power and generalization performance. In this study, TRL-FM’s abstraction of knowledge is achieved in the form of functional modules, but the overall framework is generalizable in that different approaches of acquiring abstract knowledge can be integrated into this framework.

**Electronic supplementary material:**

The online version of this article (doi:10.1186/s12859-015-0643-8) contains supplementary material, which is available to authorized users.

## Background

With the advent of high-throughput ‘transcriptomic’ technology, biomarkers measured in tissue or bodily fluids have generated a vast amount of data, from which classification models can be and have been developed to predict the early development, diagnosis, and prognosis of diseases [1]. A major challenge for class prediction tasks is that a small sample size (tens to hundreds) and a large number of variables (ranging from hundreds to several thousand) characterize most types of ‘transcriptomic’ data, like gene expression data. Classification models learned from such high-dimensional data might not generalize well nor command a strong statistical support. In addition, the heterogeneity of sample sources and experimental protocols can make discovering robust biomarkers that can predict a disease state with high fidelity very difficult.

To address these challenges a combination of multiple, but independent studies, which were designed to investigate the same biological problem, have been proposed to improve classification performance in diagnostic and prognostic models [1–4]. Two of the most common strategies for combining “transcriptomic” data for integrative modeling are via *meta-analysis* and *cross-platform data merging* [5]. In the former approach, integration occurs at the interpretive level, where results (e.g., classification accuracy, p-values, ranks, etc.) from individual studies are combined, while with the latter, integration occurs by rescaling of expression values into numerically comparable measures before the class prediction task.

A major limitation about these approaches is that they are unable to incorporate prior domain knowledge nor transfer latent biological information, which might help boost predictive performance. Studies by Ptitsyn and colleagues [6] revealed that the state (e.g., level of perturbations) of some pathways like, cell adhesion, energy metabolism, antigen presentation, and cell cycle regulation could predict metastasis progression in colorectal and breast cancer samples. Meanwhile, Huang et al. [7] suggested that pathway-based prognosis models for breast cancer performs better than a gene-based one. Thus, incorporating or transferring prior biological knowledge, such as the state of a pathway or functional associations of genes, into model generation could improve predictive performance on ‘transcriptomic’ datasets.

Ganchev and colleagues proposed a novel framework — transfer rule learning (TRL) — which leverages the concept of transfer learning to build an integrative model of classification rules from two datasets [8]. Transfer learning (TL) is the use of information learned from one task, which we call the source task, to learn another different, albeit related, task, which we call the target task [9]. Given two datasets, where one is designated as the source and the other as target, TRL builds classification rules according to two main steps. First, it learns a rule model on the source, and second, it transfers knowledge learned from the source model to seed learning of a new rule model on the target. TRL is a useful tool for integrative modeling for multiple microarray gene expression (MAGE) studies. Given two or more datasets, TRL can carry out integrative modeling in a pairwise fashion.

The TRL framework has limited capabilities. Its strategy for knowledge transfer could be improved. Generally, humans are able to recognize and apply knowledge learned from a previous task to a new task if they can align the commonalties between the two [9, 10]. For instance, skills learned from a programming language like C++, could be applied to learn a new language, like Java. Both adhere to common programming principles (e.g., both implement a “for loop”) even though the syntax can be different. Therefore, for transfer learning to be meaningful it is essential to capture the commonalities that the source and target share. TRL’s mechanism for establishing this commonality is to identify common variables between the source and target datasets. However, studies have shown that different classification models built on independent microarray datasets can contain different sets of biomarkers with little overlap. In addition, models based on different variable sets can yield similar classification performance when tested on the same validation dataset [1, 11, 12]. This means that relying solely on identical variables to establish commonality might not be enough, and therefore exploring and incorporating other means of determining variable equivalence could be vital for model performance.

Several genes, though represented by different symbols, could have something in common. For instance, they might belong to the same biological pathway or be associated to the same disease. In humans, for example, the *TP53* gene, which encodes the tumor protein p53, is known to play a key role in the activation and/or control of apoptosis [13]. Meanwhile, caspase-6, an effector caspase, which is encoded by the *CASP6* genes, cleaves to other proteins to trigger the apoptosis process [13]. Superficially, *TP53* and *CASP6* are different, but they both play a prominent role in apoptosis. TRL and several meta-analysis methods cannot capture this functional similarity or many others for integrative analysis.

We present in this paper, TRL-FM, an extension to the TRL framework, which can capture and incorporate abstract knowledge to improve integrative modeling of MAGE datasets. TRL-FM leverages *functional modules* to capture and abstract underlying commonalities, such as functional similarities, among variables across MAGE datasets. To the best of our knowledge, this is the first paper proposing the application of functional modules via knowledge transfer for integrative rule modeling of multiple gene expression datasets.

A functional module (FM) consists of a group of cellular components and their interactions that can be associated with a specific biological process. An FM can be a discrete functional entity separable from other FMs or an amalgam of various FMs with a single functional theme [14]. TRL-FM posits that biomarkers that co-occur in the same FM can possess similar predictive value, so that they can serve as proxies for each other during knowledge transfer from one dataset to another. Armed with this basis, TRL-FM should be able to recognize functionally similar, but non-identical variables (e.g., *TP53* and *CASP6* as illustrated above) to facilitate knowledge transfer.

Our goal in this study was threefold. First, to test whether FMs can be used to capture the underlying commonality among variables of different but related gene expression datasets, and are more effective when used as bridges to assist knowledge transfer than relying on identical variables. Second, to test the hypothesis that integrative modeling via the TRL-FM approach outperforms traditional models based on single gene expression data sources. Last, to evaluate and compare the classification performance of TRL-FM with traditional methods, using 21 gene expression datasets that were collected from three respective studies: one on brain cancer, one on prostate cancer, and one on a lung disease (idiopathic pulmonary fibrosis or IPF).

## Methods

**Fig. 1:**
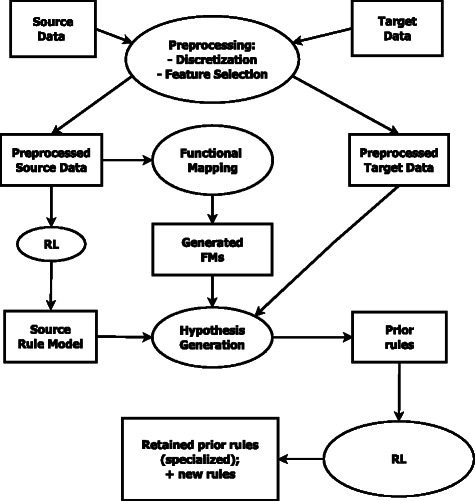
The TRL-FM framework. The framework for knowledge transfer using functional mapping and classification rules works as follows. First, use a feature selector to select relevant variables from the source and target datasets. Second, combine the selected variables into a single list and partition them into functional modules (FMs). Third, using the discovered functional modules in addition to rules induced from the source data, build a prior hypothesis of classification rules. Finally, using the prior hypothesis as a seed, learn a new classification rule model on the target data

Figure 1 depicts an overview of the TRL-FM framework. For the sake of simplicity, this framework performs transfer between two different, but related, sets of microarray data — a source and a target. However, TRL-FM, as we will show later in this article, can glean information from several sources to facilitate knowledge transfer — a strategy that is akin to receiving advice from several experts. The key steps according to the framework are as follows: First select — using a feature selection method — relevant variables from the source(s). Second, identify FMs among the selected variables. Third, using the discovered FMs, along with rules induced from the source(s) datasets; build a prior hypothesis of classification rules. Finally, using the prior hypothesis as a seed, learn a new classification rule model from the target dataset. TRL-FM is composed of four major components to execute these steps namely, feature selection via discretization, identification of functional modules, classification rule learning, and transfer learning of classification rules via functional mapping. We briefly describe these components below.

### Feature selection via discretization

MAGE datasets are comprised of hundreds or thousands of measured variables. The goal in integrative modeling is to identify and select a handful of relevant variables from among these hundreds or thousands that can accurately predict a disease state or estimate the risk of disease in an individual. The selected variables serve as building blocks for constructing classification models. Moreover, the variables in MAGE data are continuous in most cases, meaning that the variables can take an infinite number of possible values within a specified range. Continuous data pose several challenges to knowledge discovery and data mining tasks. It makes it more difficult to create compact, interpretable, and accurate classification models [15]. Several machine learning algorithms, such as decision trees [16] and rule learners [17, 18], which are used for learning classification models, handle discretized data much better [19]. It has also been shown that discretization, the process of converting a continuous variable to a discrete one, can improve the accuracy of some classifiers [20]. Integrated into the TRL-FM algorithm is a discretization method that converts continuous MAGE variables into discrete ones. After discretization, variables that have single-intervals can be filtered out since they cannot discriminate the target class. With this filtration strategy, discretization can also serve as a feature selection method.

We applied Efficient Bayesian Discretization (EBD) [19], a supervised discretization method, to discretize the input data. EBD uses a Bayesian score to discover the appropriate discretization, which guarantees optimal discretization of continuous variables from high-dimensional biomedical datasets. EBD has statistically significantly better performance than other commonly used methods for discretization [19] (see Additional file 1 for an overview of the algorithm).

### Discovering Functional Modules

Given a list of arbitrary genes, several methods can be used to identify underlying biological commonalities, which can subsequently be abstracted into domain knowledge in the form of functional modules. Biological commonality here can mean association to a common disease, function, pathway, etc. Gene set enrichment analysis (GSEA), for instance, is a popular method which is used to identify functional sets of genes associated with particular conditions of interest from ‘transcriptomic’ data. There are a plethora of GSEA methods, each with its inherent strengths and limitations [21, 22]. The focus of this paper is to highlight the utility of incorporating abstracted background knowledge to improve classification modeling, but not necessarily, to evaluate which knowledge abstraction method improves performance. To this end, we implemented a Gene Ontology (GO)-similarity-based method to identify commonalities among variables in MAGE datasets.

#### The protocol

**Fig. 2:**
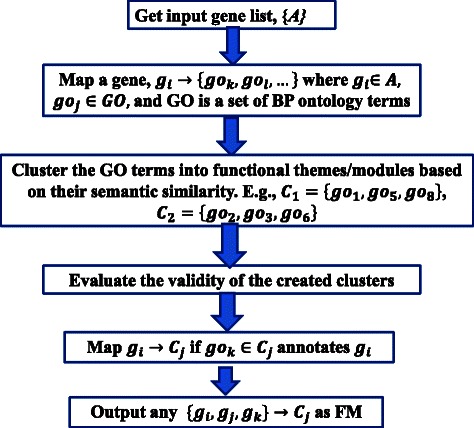
A protocol for identifying functional modules using spectral clustering and the Gene Ontology. Given an input set of genes, first map each gene to the corresponding GO term(s) that annotate(s) it according to the GO annotation database [23]. For example, if *G* denotes the set of input genes then we map each gene *g* (where *g* ∈ *G*), to the GO term *go* (where *go* ∈ *GO*) that annotates it. Here, *GO* refers to a set of biological process terms in the GO. For example, the mapping *M*(*g*_1_) = {*go*_1_, *go*_3_} means that terms *go*_1_ and *go*_3_ annotate gene *g*_1_. Second, form a set union of all GO terms that annotate at least one member of the input gene set. Third, using semantic similarity [24] as a distance measure, construct a similarity matrix among the GO terms. Fourth, with the similarity matrix as input, applied the spectral clustering algorithm [25] to group the GO terms into functionally similar clusters. Fifth, apply the Silhouette value technique [26] to estimate appropriate cluster size as well as cluster validity. Finally, map each gene *g*_*i*_ (i.e., keys of map M) to cluster *C*_*i*_ if there exist at least one term in *C*_*i*_ that annotates *g*_*i*_

Figure 2 illustrates steps to discover functional modules among an arbitrary list of genes (see Additional file 1 for additional details).

First, we mapped each gene in the input set to the corresponding GO term(s) that annotate(s) the gene, according to the GO annotation database [23]. For example, if *G* denotes the set of input genes, then we map each gene *g* (where *g* ∈ *G*), to the GO term *go* (where *g* ∈ *G*),) that annotates it. Here, *GO* refers to a set of biological process terms in the GO. For example, the mapping *M*(*g*_1_) = {*go*_1_, *go*_3_} means that terms *go*_1_ and *go*_3_ annotate gene *g*_1_. Subsequently, we formed a union of all GO terms that annotate at least one member of the input gene set. This set of GO terms served as input to the clustering phase.

Second, using semantic similarity [24] as a distance measure, we constructed a similarity matrix among the GO terms. With the similarity matrix as input, we applied the spectral clustering algorithm [25] to group the GO terms into functionally similar clusters. Subsequently, we applied the Silhouette value technique [26] to estimate appropriate cluster size as well as cluster validity.

Finally, we mapped each gene *g*_*i*_ (i.e., keys of map *M*) to cluster *C*_*i*_ if there existed at least one term in *C*_*i*_ that annotates *g*_*i*_. This enabled us to identify groups of genes that perform the same or similar functions as well as genes that perform multiple functions. Any group of genes that mapped to a particular GO cluster (e.g., {*g*_1_, *g*_2_, *g*_3_} → *C*_1_) forms a functional module.

### Classification rule learning with RL

The TRL-FM framework is driven by the rule learner (RL) [18], a classification rule learning algorithm, which has been used successfully in several classification tasks involving genomic and proteomic studies [27–30].

Given a set of training examples — a vector of variable-value pairs, including a class label —RL learns a set of IF-THEN propositional rules. RL induces rules of the form:

IF *Condition* THEN *Consequent*

where the *Condition* consists of one or more variable tests, which we also call conjuncts, and the *Consequent* denotes prediction of the target variable, also known as a class variable. Every induced rule has classification-relevant statistics associated with it. For example, let us consider the hypothetical rule below:

IF ((*gene*1 > 1680) AND (*gene*2 ≤ 28.6)) THEN (Class = Case)

$$ CF=0.98,\ P=0.007,\ TP = 56,\ FP=4 $$

where *gene*1 and *gene*2 are biomarkers with two intervals of values after discretization. We interpret the rule as follows: “when *gene*1 is up-regulated (i.e., > 1680) and *gene*2 is down-regulated (i.e., ≤ 28.6), then predict the target class as Case.” Relevant statistics are associated to each rule induced by RL. In the given example above, the ensuing statistics mean that RL induced the rule with a 98 % degree of confidence, which we call the **Certainty Factor (CF)**. Several rule evaluation functions, such as precision or Laplace estimate, are used by RL to calculate CF. **P** represents the p-value, computed by Fisher’s exact test. We define other relevant statistics as follows: **True Positives (TP)** are the number of positive examples that are correctly predicted as positive, while **False Positives (FP)** are the number of negative examples that are incorrectly predicted as positive. The TP and FP values in the example above mean that, out of 60 data instances that the rule antecedent (Conditions) matched logically, 56 were predicted correctly.

RL has several characteristics that make it particularly suitable for use in biomarker discovery studies [31]. First, unlike other knowledge discovery algorithms like artificial neural networks or support vector machines, humans can easily interpret classification models learned by RL. Second, RL is simple and flexible such that, users can leverage domain knowledge to set learning parameters *apriori* in order to improve a search in the hypothesis space. Third, RL covers rule with replacement. That is, it does not recursively partition the instance space of the training example (e.g., C4.5 [16]), nor does it eliminate training instances covered by a rule as learning proceeds (e.g., CN2 [17]), but instead it allows rules to cover overlapping regions in the instance space. Covering training instances with replacement particularly suits situations where data are scarce (e.g., microarray data), since ample data will be available to provide statistical support for newly induced rules. Fourth, RL can handle nonlinear relationships as well as hierarchical variables, such as cancer and its subtypes. Fifth, to avoid costly errors, RL can abstain (i.e., it is agnostic) from predicting a test case when it has low confidence in the accuracy of the rule [27, 31].

**Fig. 3:**
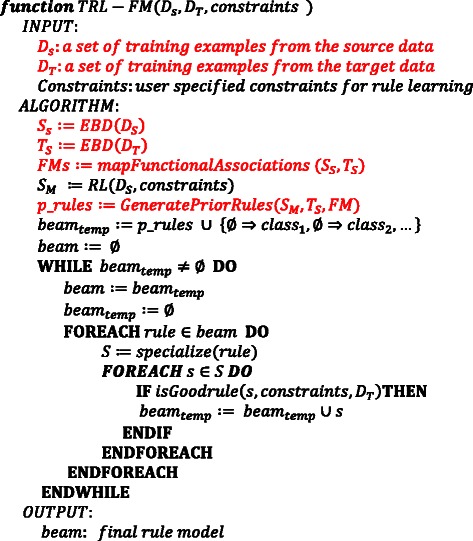
An algorithm for implementing the TRL-FM framework. This algorithm, a vast modification of the TRL algorithm [8], incorporates a subroutine (see Fig. 4) for mapping functionally related variables between the source and target data. The statements in red font are additions to the TRL algorithm

Internally, RL stores induced rules in a priority queue (aka, the beam) by sorting them according to their CF and coverage. By default, we set the beam-width (i.e., total number of rules in memory) to 1000. To construct a rule model,—a set of disjunctive rules—the RL algorithm proceeds as a heuristic beam search through the space of rules, using a general-to-specific approach [18]. First, it considers every variable as a potential predictor of the target class variable. For each discretized interval value of a marker, it creates as many rules as there are target class values. Example, for a Case/Control binary class, it will create two rules for each discretized marker value. One rule predicts Case and the other predicts Control. Second, it places an induced rule on the beam if it satisfies user-specified constraints, also known as good-rule criteria. The criteria are minimum CF value, minimum coverage, maximum false positive rate, and maximum conjuncts (i.e., the maximum number of variable-value pairs allowed in a rule antecedent). Subsequently, each rule on the beam is specialized if it satisfies the constraints. Specialization is the process whereby the rule learner successfully adds conjuncts (i.e., marker-value pairs) to the rule antecedent until the constraints are violated. The algorithm stops and outputs the set of rules on the beam if there are no more rules to specialize. This set of classification rules output by RL is referred to as a rule model. The RL algorithm has been integrated as a subroutine in the TRL-FM algorithm (see Fig. 3).

### Transfer learning of classification rules via functional mapping

The TRL-FM algorithm, illustrated in Fig. 3, implements the TRL-FM framework (see Fig. 1). In this version of the framework, transfer via functional mapping occurs between a single source and target dataset. You can modify it to include multiple source datasets or a list of biomarkers in place of the source. The latter is particularly useful when a source dataset is not readily available but markers, which can be mined from literature or gleaned from domain knowledge, are obtainable. Furthermore, this method of providing source information injects flexibility into the prior rules generation phase as it does not present the challenges, well elucidated by Ganchev et al. [31], that arise with mapping variable values — or discretized intervals — across the source and target datasets.

The algorithm accepts as inputs the source and target datasets, including user specified constraints (i.e., minimum CF, minimum coverage, inductive strengthening, and maximum conjuncts) for RL. EBD discretizes the input dataset if they contain continuous variables. Next, FMs are discovered among the selected variables from EBD to facilitate the transfer of knowledge for learning a model on the target dataset. Knowledge transfer occurs via the formulation of prior hypothesis (i.e., a set of rules), which is used to seed learning of the target model.

**Fig. 4:**
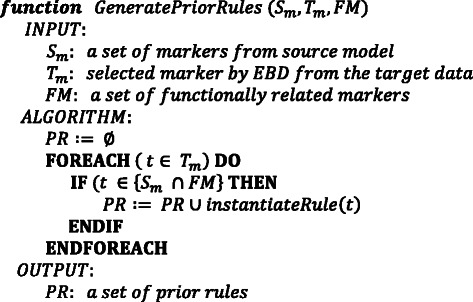
An algorithm for generating prior rules for seeding learning of a rule model. This algorithm, a subroutine within the TRL-FM framework, leverages information from domain knowledge, through functional modules, to instantiate prior rules to seed learning on the target data

The source dataset is first analyzed using RL. The rule model learned on the source, in combination with the FMs, is used through the *GeneratePriorRules* function (see Fig. 4) to formulate a prior hypothesis, which is used to seed learning of a new rule model on the target. Using the FM as a bridge, the function instantiates prior rules as follows:

1. For a particular functional module, *FM*_*k*_, select a variable, *a*_*Tj*_, for rule instantiation if the condition below holds:

$$ \left({a}_{Tj}\in " Set2"\right) \wedge \left(\left\{{a}_{Si},{a}_{Tj}\right\}\in F{M}_k\right)\wedge \left({a}_{Si}\in " Set1"\right). $$

2. Build a prior rule “structure” as follows:

$$ IF\ \left({a}_{Tj}=?\right) THEN\ \left( Class=?\right) $$

3. For every selected variable, instantiate a prior rule structure with all discrete ranges of values and all class values. For example, if the discretized ranges of values for a marker, ***a***_***Tj***_ **,** are LOW and HIGH and the target class values are Case and Control, then the instantiated rules become:

$$ IF\ \left({a}_{Tj}= LOW\right) THEN\ \left( Class= Case\right) $$

$$ IF\ \left({a}_{Tj}= HIGH\right) THEN\ \left( Class= Case\right) $$

$$ IF\ \left({a}_{Tj}= LOW\right) THEN\ \left( Class= Control\right) $$

$$ IF\ \left({a}_{Tj}= HIGH\right) THEN\ \left( Class= Control\right) $$

Subsequently, the instantiated rules are loaded onto the beam and learning proceeds as a heuristic beam search in a typical RL fashion, as described above. In the specialization step (see Fig. 3), through the *specialize()* function, all non-redundant patterns obtained by adding a single variable-value pair to a rule’s antecedent are considered. Note that all induced rules, including the prior rules, that do not satisfy the “good rule” criteria are pruned away (i.e., discarded). A rule is “good” if it satisfies the user-specified constraints.

## Results and discussion

### Experiments

To test the feasibility of TRL-FM as a viable tool for integrative modeling of MAGE datasets we applied the framework to learn classification rule models using publicly available datasets. The goals of the experiments were threefold. First, to ascertain TRL-FM’s ability and flexibility in capturing abstract biological knowledge from source datasets in order to facilitate transfer learning. Second, to evaluate the classification performance of models built by TRL-FM, and how it compares with traditional methods built on single source datasets. Last, compare the performance of integrative modeling via the TRL-FM approach with meta-analysis and cross-platform data merging methods.

#### Datasets

Table 1 provides details of the three example MAGE datasets that we used for the experiments. Each example contained 7 microarray studies of two-group comparison (i.e., case vs control). The datasets were collected from three studies: a brain cancer study, a prostate cancer study, and an IPF study. These datasets particularly suit the goals of our experiments and the utility of integrative modeling of MAGE datasets because, (1) they are publicly available, (2) they have been used extensively to test experiments in several integrative modeling studies, and (3) they were generated using diverse microarray platforms. Testing the flexibility of TRL-FM with datasets generated using diverse platforms is essential since TRL and many meta-analysis methods require identical platforms and variables for integrative modeling. That is, TRL-FM avoids the critical and often challenging task of mapping features (e.g., gene names) across disparate platforms for integrative modeling.

**Table 1:** Experimental data sources. Sources of data for experiments and their descriptions

Disease	Author	Year	Platform	Sample Size (Cases/Controls)	Source
Prostate Cancer	Singh	2002	HG-U95Av2	102 (52/50)	www.broad.mit.edu
	Lapointe	2004	cDNA	103 (62/41)	GSE3933
	Wallace	2008	HGU133A2	89 (69/20)	GSE6956
	Nanni	2006	HG-U133A	30(23/7)	GSE3868
	Varambally	2005	HG-U133 Plus 2	13(7/6)	GSE3325
	Welsh	2001	HG-U95A	34(25/9)	public.gnf.org/cancer
	Yu	2004	HG-U95Av2	83(65/18)	GSE6919
Brain Cancer	Freije	2004	HG-U133A,B	85 (59/26)	GSE4412
	Phillips	2006	HG-U133A,B	100 (76/24)	GSE4271
	Sun	2006	HG-U133 Plus 2	100 (81/19)	GSE4290
	Petalidis	2008	HG-U133A	58 (39/19)	GSE1993
	Gravendeel	2009	HG-U133 Plus 2	175(159/16)	GSE16011
	Paugh	2010	HG-U133 Plus 2	42(33/9)	GSE19578
	Yamanaka	2006	Agilent	29(22/7)	GSE4381
Lung Disease Studies (IPF)	Pardo	2005	Codelink	24(13/11)	GSE2052
	Yang	2007	Agilent 43 K	29(20/9)	GSE5774
	Konishi	2009	Agilent 4x44K	38(23/15)	GSE10667
	KangA	2011	Agilent 4x44K	63(52/11)	Dr. Kaminski
	KangB	2011	Agilent 8x60K	96(75/21)	Dr. Kaminski
	Larsson	2008	HG-U133 Plus 2	12(6/6)	GSE11196
	Emblom	2010	cDNA	58(38/20)	GSE17978

#### Experimental Design

Our task was to build a classification rule model that can classify normal tissue versus diseased tissue from same organ, e.g., to distinguish normal prostate tissue from prostate cancer. We designed our experiments to evaluate if knowledge learned from datasets of the same MAGE example set (i.e., same organ of origin, like IPF) can be transferred to enhance learning of a classification model on a new dataset. For each example set of the experimental datasets, consider a set of *n* datasets, *D* = {*D*_1_, *D*_2_, …, *D*_*n*_}, where *D*_*i*_ represents the *i*th dataset. Within a set, each dataset, *D*_*i*_, in turn was set as target, while the rest, {*D* − *D*_*i*_}, were designated as source data for knowledge transfer. Guided by the TRL-FM framework (see Fig. 1), classification rule models were generated from the source datasets—one model per dataset. With this approach, *n* number of TRL-FM experiments can be performed within a set, so in all, we executed 21 (i.e., 3 X 7) experiments. This study design strategy was necessary to test the notion that knowledge transfer from multiple sources will more likely improve learning on the target.

#### Evaluation

We used the area under the Receiver Operative Characteristic curve (AUC) [32] to evaluate the predictive efficacy of TRL-FM. For each experiment, we measured the mean of the AUC on 10-fold cross-validation. In addition, we also estimated the performance of TRL-FM when each FM was used solely as a bridge for transfer. The rationale for this strategy was to ascertain whether particular functional themes improve the baseline performance (or not). We could have experimented with different combinations of the FMs to determine which particular group(s) optimizes learning. However, for *n* FMs, such an approach will yield approximately $$ \left(\begin{array}{c}\hfill n\hfill \\ {}\hfill 1\hfill \end{array}\right)+\left(\begin{array}{c}\hfill n\hfill \\ {}\hfill 2\hfill \end{array}\right)+\dots +\left(\begin{array}{c}\hfill n\hfill \\ {}\hfill n\hfill \end{array}\right) $$ models, which is computationally intractable—in the advent of high-performance computing, this process can be automated. For the sake of simplicity, we instead experimented with an ensemble of all FMs.

In addition, we compared the performance of TRL-FM over TRL and RL (baseline). Note that the TRL framework is constrained with a single dataset as source, while TRL-FM extracts knowledge from multiple sources, via functional mapping. This means that for evaluating the TRL experiments, for every *i*th dataset that was designated as target, the rest of the datasets in the same study (e.g., IPF) in turn had to be set as source.

Finally, we compared the performance of our methods with traditional algorithms for single source datasets namely, Support Vector Machines (SVM), Linear Discriminant Analysis (LDA), Random Forest (RF), C4.5, Naïve Bayes (NB), and Penalized Logistic Regression (PLR). Using the same metric (i.e., AUC on 10-fold cross-validation), we evaluated the classification performance of these methods on the raw datasets as well as cross-study integration via meta-analysis and data merging. Several methods have been proposed for microarray data merging via meta-analysis and cross-platform data merging [5, 33], but for the sake of brevity and demonstration purposes, we adopted the adaptively weighted (AW) Fisher method [34], while we applied COMBAT [35], which uses an empirical Bayes method to adjust for batch effect across multiple gene expression studies before merging.

### Identification of functional modules

For each round of TRL-FM experiments, we identified a set of FMs from the list of relevant source variables. In all, 21 sets of FMs were generated, since each dataset, in turn, was set as target in each round of experiment. To simplify the rest of this discussion, we have randomly selected and present one FM table from each disease study — Tables 2, 3, and 4. We provide the rest as supplementary data (see Additional file 2).

**Table 2:** FMs for target, Petalidis. Functional modules to facilitate functional mapping to target (Petalidis) variables from sources (Freije, Gravendeel, Paugh, Phillips, Sun, Yamanaka) variables

Clusters	GO Functional Theme	Markers
FM1	DNA repair	*ADCY3, ADCY7, ALDH6A1, BLVRA, CSTF2T, DHX9, DNASE1L1, MCM2, MRE11A, STRAP, USP47*
FM2	Apoptotic processes	*ADAMTSL4, ADORA1, ARAF, CASP5, CTSL2, FKTN, MCM2, MRE11A, P2RX1, RPS6KA3, SGPL1, STRN, TEX261, TRIAP1, USP47, VEGFA*
FM3	Regulation of protein phosphorylation	*ADCY3, ADCY7, ADORA1, ARAF, CDC37, DVL1, MRE11A, PPP2R5A, VEGFA*
FM4	Cell differentiation	*ACTG1, ALDH6A1, AP2B1, CRB1, DVL1, EFNB2, IFRD1, JAG1, MGP, MYO10, NHLH2, PBX1, RPS6KA3, RYR1, SGPL1, STRN, VEGFA, ZIC3, ZMYM3*
FM5	Transport	*ABCC10, ADCY3, ADCY7, ADORA1, AP2B1, CDC37, LRMP, MSR1, MYO10, P2RX1, PDIA4, RYR1, VEGFA*
FM6	Signal transduction	*ADCY3, ADCY7, ADORA1, AP2B1, ARAF, CD97, CSNK1G1, CXCL6, DVL1, EFNB2, IGFBP2, JAG1, LANCL1, NDST1, P2RX1, PPP2R5A, RPS6KA3, SGPL1, STC1, STRN, VEGFA*
FM7	Cell proliferation	*IGFBP2, JAG1, MSR1, MYO10, PBX1, RPS6KA3, USP47, VEGFA*
FM8	Response to glucose stimuli	*ADCY3, ADCY7, CTSL2, CYP2E1, IGFBP2, JAG1, NDST1, P2RX1, RPS6KA3, RYR1, STC1, STRAP, VEGFA*
FM9	Toll-like receptor signaling	*RPS6KA3*
FM10	Transcription	*ADCY7, BTAF1, DAZL, DVL1, FBN1, JAG1, MRE11A, NHLH2, NKRF, PBX1, RPS6KA3, STRAP, USP47, VEGFA, ZIC3, ZMYND11, ZNF187*
FM11	Response to stress	*ACTG1, ADORA1, CASP5, CD97, CTSL2, CXCL6, DHX9, IGFBP2, LDHA, MRE11A, NDST1, NPEPPS, P2RX1, RPS6KA3, RYR1, STC1, TRIAP1, USP47, VEGFA*

**Table 3:** FMs for target, KangA. Functional modules to facilitate functional mapping to target (KangA) variables from sources (Emblom, KangB, Konishi, Larsson, Pardo, Yang) variables

Clusters	GO Functional Theme	Markers
FM1	Regulation of kinase activity	*CBS, FCER1A, THY1*
FM2	Notch signaling	*BAI2, CNTNAP2, HEY1, PKIG*
FM3	Cell junction assembly	*ASPN, CBS, CNTNAP2, HEY1, KLK7*
FM4	Cell adhesion	*CDH2, CNTNAP2, THY1*
FM5	T cell receptor signaling pathway	*ASPN, CDH2, FCER1A, HEY1, THY1*
FM6	Brain development	*BAI2, CBS, CNTNAP2*
FM7	Protein homooligomerization	*DPYSL3, MPP6*
FM8	Pyrimidine nucleobase catabolic process	*CBS, DPYSL3, THY1*
FM9	Transcription	*HEY1, HR, PKIG, FCER1A*
FM10	Transsulfuration	*CBS*
FM11	Muscle cell differentiation	*CDH2, HEY1, SRD5A1*
FM12	Sex determination	*CBS, CNTNAP2, SRD5A1*
FM13	Superoxide metabolic process	*CBS*
FM14	Cellular protein localization	*CNTNAP2*

**Table 4:** FMs for target, Lapointe. Functional modules to facilitate functional mapping to target (Lapointe) variables from sources (Nanni, Singh, Varambally, Wallace, Welsh, Yu) variables

Clusters	GO Functional Theme	Markers
FM1	Cardiac and urinary organ morphogenesis	*ACTL6A, ANXA2, ERG, FZD7, GATA3, GATM, JUND, NFATC3, SOX9, WHSC1*
FM2	Lipid metabolism	*ABCA2, AMACR, C3, GATA3, GATM, LEPR, NFATC3*
FM3	Regulation of chemokine production	*C3, DARC, GATA3, SCGB1A1, SOX9*
FM4	Histone acetylation and methylation	*ACTL6A, C3, GATA3, MUC1, NELL1, PRKCB, SOX9*
FM5	Signal transduction	*ACTL6A, ADCY2, BCAM, C3, CCL1, DARC, DYNLT1, ERG, FZD7, GATA3, GDI1, GJA1, KCNN4, LEPR, MAP3K14, MUC1, PRKCB, RCAN2, SCGB1A1, SNAI2, SOX9, USP33, WIF1*
FM6	Chemotaxis	*ABCA2, CCL1, GATA3, GATM, GDI1, JUND, SCGB1A1*
FM7	Transcription	*ACTL6A, BCAS2, ERG, GATA3, GATM, JUND, NFATC3, NFYB, POLR2H, PRKCB, RPS29, WHSC1*
FM8	Regulation of transcription	*ABCA2, ACTL6A, ETV5, FOSB, FZD7, GATA3, JUND, MUC1, NFATC3, NFYB, POLR2H, PRKCB, SCGB1A1, SNAI2, SOX9, TCEAL4, WHSC1*
FM9	Translation	*DNAJC11, EEF2, ERG, MUC1, POLR2H, PRKCB, RPS29, USP33*
FM10	Cellular response to cytokines	*ANXA2, DARC, FOSB, FZD7, GATA3, JUND, NFATC3, PRKCB, SOX9*

Table 2 shows the functional modules that facilitated rule transfer when we set Petalidis (brain cancer) as the target dataset. Similarly, Tables 3 and 4 represent functional modules when KangA (IPF) and Lapointe (prostate cancer) were set as targets, respectively. Observe that the number of functional modules are not necessarily the same for all target data. KangA, for example, had 14, while Lapointe contained 10. This means that the former and latter had 14 and 10 FMs, respectively, that were functionally homogenous — that is, had average Silhouette values of at least 0.5.

We made three observations from the functional modules. First, almost all of the functional themes were composed of more than one gene. Second, some genes were multi-functional. That is, they were associated with more than one different functional theme. In Table 2, for instance, *ADCY3* (adenylate cyclase 3) was associated with DNA repair, protein phosphorylation, transport, and response to glucose stimuli. We made a similar observation in Table 3, where *CBS* (cystathionine beta synthase) was associated with some metabolic processes, brain development, and the regulation of kinase activity. Lastly, most of the discovered functional themes like signal transduction, apoptotic processes, cell differentiation, cell proliferation, and many others, are associated with the hallmarks of cancer [6, 36].

The biological information revealed from these observations obtained using the TRL-FM style to capture, abstract, and formulate propositional rules for knowledge transfer could be essential for algorithm and model development for integrative modeling of MAGE datasets. Normally, for symbolic data mining algorithms like RL, the interestingness criteria (i.e., how good a rule is) for a newly induces rule is evaluated by objective methods like *confidence* (e.g., positive predictive value) and *support* (e.g., the probability that a pattern will occur). Other subjective methods, which leverage background knowledge or an expert opinion, have also been proposed to define explicit criteria for rule interestingness [37]. Prior knowledge, for instance, when gleaned from functional modules, literature, and/or a domain expert, could be incorporated into classification rule induction to contribute, subjectively, to the evaluation of how good an induced rule is. However, while the incorporation of FMs into the TRL-FM framework facilitated the mapping of variables across source(s) and target datasets, the biological knowledge contained in them did not explicitly contribute to rule confidence within the rule-induction engine of the framework. It rather affected the learning bias of the algorithm by seeding the search with prior information. That is, instead of learning from scratch it starts learning from a point in the search space that is presumably closer to the target solution.

### RL vs TRL vs TRL-FM

With Tables 5 and 6, we compare and contrast the performance TRL-FM with its predecessors, RL and TRL, on three datasets (Petalidis, KangA, and Lapointe), one from each disease type. Similarly, we provide results for the rest of the datasets as supplementary data (see Additional file 3 and Additional file 4). Tables 5 and 6 show the performances of classification rule models learned with and without TRL-FM (i.e., TRL-FM vs baseline RL) and TRL, respectively (i.e., TRL vs baseline RL). Table 7 (see Additional file 5 for detailed results) summarizes the overall performances of the three algorithms, including the other traditional method (i.e., SVM, LDA, RF, C4.5, NB, and PLR), on all datasets. In addition, Tables 5 and 6 provides information for sources of knowledge transfer. That is FMs, including their union, for TRL-FM, and for TRL, every possible source within a disease type. In the summary table, we show results for TRL-FM using union FMs, while for TRL; the AUC from the best performing source is displayed. For example, with Petalidis as target, the best performing source was Gravendeel (see Additional file 4 for details).

**Table 5:** Comparison of TRL-FM with baseline RL. AUCs when RL (baseline) and TRL-FM are applied to build a classification rule model on three datasets, Petalidis (brain), KangA (IPF), and Lapointe (prostate). For TRL-FM, the FMs are the medium through which knowledge transfer occurs. “Union” is an ensemble of all FMs. The mean and the standard error of the mean (SEM) for the AUC of a dataset was obtained by 10-fold cross-validation

Dataset	Petalidis	KangA	Lapointe
	AUC (SEM)	AUC (SEM)	AUC (SEM)
Baseline	0.83 (0.06)	0.86 (0.07)	0.93 (0.03)
FM1	0.82 (0.07)	**0.93 (0.05)**	0.87 (0.04)
FM2	**0.89 (0.07)**	**0.92 (0.05)**	0.88 (0.04)
FM3	**0.89 (0.07)**	0.85 (0.07)	**0.96 (0.02)**
FM4	**0.88 (0.06)**	**0.89 (0.07)**	0.90 (0.03)
FM5	**0.84 (0.08)**	0.81 (0.07)	0.90 (0.03)
FM6	**0.85 (0.06)**	**0.86 (0.07)**	**0.94 (0.02)**
FM7	0.81 (0.07)	0.82 (0.07)	**0.95 (0.03)**
FM8	**0.86 (0.06)**	**0.93 (0.05)**	0.92 (0.03)
FM9	0.81 (0.07)	0.86 (0.07)	0.88 (0.04)
FM10	**0.84 (0.08)**	0.86 (0.07)	0.89 (0.03)
FM11	**0.89 (0.07)**	0.82 (0.07)	
FM12		0.86 (0.07)	
FM13		**0.93 (0.05)**	
FM14		**0.93 (0.05)**	
Union	**0.91 (0.06)**	**0.97 (0.03)**	**0.97 (0.02)**

For each dataset, positive transfer is shown in bold font, while underlined AUCs denote negative transfer

**Table 6:** Comparison of TRL with baseline RL. AUCs when RL (baseline) and TRL are applied to build a classification rule model on three datasets, Petalidis (brain), KangA (IPF), and Lapointe (prostate). SRC means the source dataset (e.g., for target Petalidis, SRC1 is Freije, see Additional File 3). The mean and the standard error of the mean (SEM) for the AUC of a dataset was obtained by 10-fold cross-validation

Dataset	Petalidis	KangA	Lapointe
	AUC (SEM)	AUC (SEM)	AUC (SEM)
Baseline	0.83 (0.06)	0.86 (0.07)	0.93 (0.03)
SRC1	0.82 (0.05)	0.86 (0.07)	0.93 (0.03)
SRC2	**0.88 (0.07)**	0.86 (0.07)	0.89 (0.05)
SRC3	0.81 (0.07)	**0.93 (0.05)**	0.90 (0.03)
SRC4	0.78 (0.06)	0.86 (0.07)	0.93 (0.03)
SRC5	**0.85 (0.05)**	0.86 (0.07)	0.91 (0.04)
SRC6	0.81 (0.07)	0.86 (0.07)	0.91 (0.04)

For each dataset, positive transfer is shown in bold font, while underlined AUCs denote negative transfer

**Table 7:** Comparison of classification performance of all classifiers on all datasets. Comparison of classification performance (AUC) among selected machine learning methods namely, Support Vector Machines (SVM), Linear Discriminant Analysis (LDA), Random Forest (RF), C4.5, Naïve Bayes (NB), Penalized Logistic Regression (PLR), as well as RL (baseline), TRL, and TRL-FM on all datasets. Note that for TRL, the AUC for the highest performing source is shown, while for TRL-FM, the medium of knowledge transfer is the union of all FMs. In addition, the average (AVG) AUC performances, including average standard error of the mean, for each classifier across the entire datasets are provided (see Additional File 5 for detailed results)

Dataset	SVM	LDA	RF	C4.5	NB	PLR	RL	TRL	TRL-FM
Emblom	1.00	1.00	1.00	0.98	0.96	0.98	0.97	0.97	0.94
Freije	0.74	0.72	0.72	0.73	0.82	0.76	0.76	0.78	0.80
Gravendeel	0.52	0.59	0.59	0.53	0.63	0.56	0.49	0.49	0.59
KangA	0.93	0.86	0.86	0.79	0.94	0.90	0.86	0.93	0.97
KangB	0.91	0.87	0.87	0.87	0.91	0.95	0.83	0.91	0.95
Konishi	0.90	0.68	0.68	0.74	0.90	0.90	0.78	0.83	0.95
Lapointe	0.96	0.91	0.91	0.94	0.97	0.96	0.93	0.93	0.97
Larsson	0.33	0.67	0.67	0.58	0.67	0.67	0.75	0.75	1.00
Nanni	0.70	0.61	0.61	0.44	0.57	0.65	0.54	0.54	0.64
Pardo	0.83	0.85	0.85	0.63	0.80	0.88	0.85	0.90	0.95
Paugh	0.48	0.45	0.45	0.43	0.50	0.45	0.51	0.52	0.54
Petalidis	0.75	0.71	0.71	0.69	0.80	0.80	0.83	0.88	0.91
Phillips	0.73	0.70	0.70	0.66	0.75	0.80	0.66	0.73	0.78
Singh	0.89	0.90	0.90	0.89	0.88	0.91	0.89	0.89	0.93
Varambally	1.00	0.92	0.92	0.67	1.00	1.00	0.83	1.00	1.00
Wallace	0.82	0.85	0.85	0.76	0.81	0.87	0.76	0.81	0.84
Welsh	0.94	0.66	0.66	0.79	0.93	0.94	0.92	0.95	0.93
Yamanaka	0.57	0.57	0.57	0.56	0.71	0.56	0.50	0.50	0.79
Yang	0.69	0.51	0.51	0.89	0.57	0.73	0.94	0.94	0.89
Yu	0.94	0.93	0.93	0.80	0.97	0.94	0.88	0.90	0.93
AVG AUC	0.77	0.74	0.74	0.71	0.80	0.81	0.77	0.80	0.86
AVG SEM	0.06	0.07	0.07	0.07	0.06	0.05	0.07	0.06	0.04

The goal of transfer learning is to improve the learning performance on the target task. Positive transfer occurs when the transferred knowledge from the source improves classification performance on the target, while negative transfer is the reduction of performance on the target after knowledge transfer. The AUCs (in Tables 5 and 6) with bold font denote positive transfer, while those resulting from negative transfer are underlined. Generally, learning with TRL-FM yielded more positive transfers than TRL. In addition, transfer with the union of FM usually produced positive transfer, while with TRL; you have to experiment with all available sources to determine the best possible transfer. Thus, while the outcomes of integrative modeling with the TRL-FM framework will most likely lead to positive transfer, currently, it cannot be estimated, a priori, which particular source from the same set of data will lead to a positive transfer if you use the TRL framework. Furthermore, a Mann–Whitney paired-sample signed rank test with a significance level *α* = 5 % showed that transfer with TRL-FM statistically significantly improves the baseline than even the best TRL (see Table 8).

**Table 8:** Pairwise significance test for classification performance among all methods. A Mann–Whitney paired-sample signed rank test with significance level *α* = 5 %. P-values were adjusted with the Benjamini Hochberg method [45]

Method	SVM	LDA	RF	C4.5	NB	PLR	RL	TRL
LDA	0.1230							
RF	0.1230							
C4.5	**0.0386**	0.0943	0.0943					
NB	0.3453	**0.0076**	**0.0076**	**0.0035**				
PLR	0.0737	**0.0043**	**0.0043**	**0.0043**	0.6280			
RL	0.3473	0.6825	0.6825	**0.0137**	0.1151	0.0700		
TRL	0.6924	0.0648	0.0648	**0.0017**	0.8666	0.6825	**0.0076**	
TRL-FM	**0.0094**	**0.0006**	**0.0006**	**0.0002**	**0.0094**	**0.0217**	**0.0017**	**0.0052**

Significant p-values are displayed in bold font

These results highlight the impact of FMs on the induction of a rule model. As we observed, no particular functional theme (s) consistently improved classification performance across all target datasets. That is, there was no direct correlation between functional themes and positive (or negative) transfer. However, what became clear was that an ensemble of the FMs, most often than not, resulted into positive transfer. The reason for this improvement could be that an aggregate of FMs widens the space of relatedness among variables of the source and target datasets. The intuition here is that, the more related two domains are, the better the learning performance of transfer learning. In addition, when snippets of information from the FMs are fused together, potential errors inherent in knowledge transfer via individual FMs can be alleviated. Meanwhile, results from other studies support our take that a combination of FMs (e.g., group of pathways), more often than not, improves performance for integrative analysis of genomic data [7, 38].

Furthermore, since TRL-FM is able to capture and abstract underlying domain knowledge, in the form of functional modules, it is able to go a step further to ask the question whether two or more identically different biomarkers have any commonality among them. This capability of TRL-FM makes it more intelligent and effective for transfer learning than TRL. That is, TRL-FM can facilitate knowledge transfer among MAGE datasets that have different variable symbols, as long as the variables can be mapped to a common biological function (s). For example, in the transfer of classification rules from the Larsson to KangA data (all from the IPF set), TRL is unable to transfer knowledge because the set of variables (*MPP6*, *PKIG*) used to build the source model does not overlap the set of variables (*ASPN*, *FMO5*, *MMP11*, *IL13RA2*) which the target model incorporates. TRL-FM, on the other hand, is able to transfer knowledge because of the association of *PKIG* (from source model) and *ASPN* (from target model) to cell signaling (Table 3). Another example here is the transfer of classification rules from the Nanni dataset to Lapointe dataset—both of the prostate set. As in the previous case, the set of variables (*CCL1*, *MUC1*, *ATOX1*, *BCAM*, *BAT3*) contained in the source model, does not overlap with that (*MYL6*, *ADCY2*, *GJA1*, *TCEAL4*, *PARG*, *MTMR7*, *SEC23A*, *ACTA2*, *COQ7*, *SNAI2*, *MAP3K14*) incorporated in the target model. Nevertheless, TRL-FM was able to use functional mapping via FMs to instantiate prior rules for seeding learning on the target using *ADCY2*, *GJA1*, *SNAI2*, and *MAP3K14* due to their functional association with *MUC1* — signal transduction and regulation of transcription (Table 4). Using the TRL framework, which requires the recognition of identical variables across the same source and target, this knowledge transfer could not have occurred.

### Comparison with other methods

The results displayed with Tables 7 and 8 indicate that integrative modeling via the TRL-FM approach statistically significantly improves traditional models based on single source datasets. The advantage TRL-FM has over the traditional models is that it is able to pool information, via transfer learning and functional mapping, from other data sources to enhance model development. Combining information from different source datasets, via biological knowledge bases, for model building may reduce inherent noise, which hampers predictive performance. Thus for transcriptomic datasets, which are mostly characterized by small sample sizes and large variable sets, integrative modeling, via the TRL-FM approach, is a viable mechanism to boost predictive power and generalization performance.

Table 9 (see Additional File 6 for further details) shows the performance of all non-transfer learning based classifiers on the datasets after integration with meta-analysis. The results indicate that there were no significantly clear improvements in performance as compared to transfer learning. What is more, in Table 10 we compare the average classification performance within each disease type (e.g., brain cancer) versus the performance when disease specific datasets were merged, into one data matrix, via meta-analysis and batch effect removal. Classification performance on the meta-analysis inspired dataset was not significantly different from average performance per disease type. However, we observed a significant reduction in performance when disease specific datasets were merged via removal of systematic bias. This result is not too surprising as a similar observation was made in a related study [3]. It is most likely that the method could not handle, effectively, the heterogeneity inherent across the different studies.

**Table 9:** Comparison of classification performance of all non-transfer rule learning classifiers on post meta-analysis datasets. Using the AW [34] meta-analysis method only biomarkers with statistically significant effect size within a particular disease type are used for a class prediction task (see Additional File 6 for further details)

Dataset	SVM	LDA	RF	C4.5	NB	PLR	RL
Emblom	1.00	1.00	1.00	0.99	0.99	0.99	0.96
Freije	0.77	0.74	0.74	0.71	0.72	0.79	0.73
Gravendeel	0.50	0.73	0.73	0.59	0.69	0.67	0.49
KangA	0.82	0.72	0.72	0.86	0.93	0.96	0.86
KangB	0.94	0.89	0.89	0.85	0.94	0.93	0.83
Konishi	0.88	0.58	0.58	0.77	0.88	0.87	0.80
Lapointe	0.95	0.92	0.92	0.91	0.95	0.95	0.91
Larsson	0.33	0.33	0.33	0.67	0.42	0.67	0.75
Nanni	0.56	0.68	0.68	0.55	0.72	0.66	0.75
Pardo	0.88	0.88	0.88	0.78	0.83	0.88	0.80
Paugh	0.57	0.45	0.45	0.65	0.51	0.66	0.52
Petalidis	0.86	0.66	0.66	0.75	0.82	0.79	0.84
Phillips	0.75	0.83	0.83	0.68	0.80	0.81	0.64
Singh	0.92	0.86	0.86	0.84	0.87	0.90	0.92
Sun	0.73	0.66	0.66	0.66	0.73	0.73	0.69
Varambally	0.75	0.92	0.92	0.79	0.92	1.00	0.83
Wallace	0.81	0.82	0.82	0.77	0.76	0.81	0.70
Welsh	0.98	0.80	0.80	0.85	0.98	0.91	0.92
Yamanaka	0.63	0.42	0.42	0.79	0.71	0.70	0.61
Yang	0.74	0.51	0.51	0.90	0.71	0.80	0.94
Yu	0.91	0.92	0.92	0.87	0.92	0.95	0.90
AVG AUC	0.78	0.73	0.73	0.77	0.80	0.83	0.78
AVG SEM	0.06	0.07	0.07	0.07	0.06	0.06	0.07

**Table 10:** Comparing average performance per disease type to merged datasets per disease type. This table shows the average classification performance per disease type as compared to merged datasets per disease type. In the dataset column, Avg denotes average, MM denotes merged by meta-analysis, and M means merged by cross-platform data merging

Dataset	SVM	LDA	RF	C4.5	NB	PLR	RL	TRL	TRL-FM
Average performance per disease type									
Avg_brain	0.67	0.66	0.66	0.64	0.73	0.69	0.66	0.68	0.76
Avg_ipf	0.80	0.78	0.78	0.78	0.82	0.86	0.85	0.89	0.95
Avg_prostate	0.89	0.83	0.83	0.76	0.88	0.90	0.82	0.86	0.89
Merged per disease type by meta-analysis									
MM_brain	0.67	0.70	0.70	0.69	0.70	0.69	0.67	*	*
MM_ipf	0.88	0.88	0.88	0.85	0.74	0.88	0.81	*	*
MM_prostate	0.89	0.84	0.84	0.81	0.70	0.85	0.76	*	*
Merged per disease type by batch effect removal									
M_Brain	0.50	0.51	0.51	0.48	0.53	0.51	0.54	*	*
M_IPF	0.67	0.63	0.63	0.60	0.63	0.64	0.68	*	*
M_Prostate	0.53	0.53	0.53	0.53	0.53	0.55	0.59	*	*

*denotes that transfer learning methods were not evaluated. Currently, TRL and TRL-FM cannot be applied to cross-domain studies (i.e., transfer from one disease type to another)

Overall, integrative modeling, via the transfer rule learning and functional mapping approach performs better as compared to methods inspired by meta-analysis and cross-platform data merging. Highlights from the results suggest that in predictive model design it might be better to focus on sub populations or individual studies as opposed to merging independent studies into one data matrix. In addition, while meta-analysis is a viable approach for integrative MAGE analysis, it cannot transfer information among datasets in order to boost performance, and more so robust differential expression does not necessarily translate into high predictive power.

### Limitations and future work

Though our preliminary empirical results suggest that the TRL-FM framework is sound, we have identified potential limitations and several avenues for future work. First, in building FMs we relied only on the GO as the information source. Although the results are promising, relying on GO as the only source from which to extract domain knowledge might limit the knowledge base of the framework for generating prior rules for transfer. Future work could expand this knowledge base by exploring and incorporating other methods of eliciting domain knowledge for transfer learning. For instance, the GO driven functional mapping module could morph into a lookup table, which would integrate information from other sources like Online Mendelian Inheritance in Man (OMIM) [39], the Kyoto Encyclopedia of Genes and Genomes (KEGG) [40], MSigDB [41], BioCarta [42], Reactome [43], and/or the Pharmacogenomics Knowledge Base (PharmGKB) [44]. This might boost prior rule generation through a more confident relational mapping between source(s) and target. Second, in particular instances, both TRL and TRL-FM frameworks yield negative transfer. Negative transfer, which is akin to giving “bad advice”, can be detrimental to model generation for diagnostic and prognostic studies. A future study could investigate the relative risks of transfer of classification rules, which would help make an immense contribution to the question of “when to transfer,” an open research problem in the transfer learning community. Last, after incorporating the above propositions into a more robust and well-refined TRL-FM framework, the feasibility of pan-cancer transfer of classification rules for integrative modeling could be explored.

## Conclusions

In this paper, we develop and evaluate a novel TRL-FM framework that extends existing classification rule-learning methods to use abstract domain knowledge to facilitate integrative modeling of multiple types of gene expression data. Empirical results from this study highlight a couple of key points. First, the results from our comprehensive experiments conducted in this paper lend strong support to our hypothesis that the TRL-FM approach can statistically significantly outperform TRL, including traditional models based on single gene expression data sources. Second, TRL-FM’s ability to leverage functional modules to capture the relatedness among source and target variables is more intelligent, effective, and biologically intuitive than TRL’s reliance on variable overlaps, which can be superficial and uninformative. Third, integrative modeling, via the TRL-FM framework leads to better performance than other integrative analysis approach, like meta-analysis, which cannot transfer vital information from one dataset to another. Last, the TRL-FM framework, when extended and refined, can serve as a viable alternative and/or complementary methodology for integrative modeling of multiple ‘transcriptomic’ datasets.

## Additional files

Additional file 1:
**Supplementary methods.** Additional information on discretization and semantic similarity.
Additional file 2:
**Table of Functional modules.** A list of functional modules that facilitated knowledge transfer for classification rule modeling on respective target datasets.
Additional file 3:
**Table complete TRL-FM results.** Classification performance of a TRL-FM model on all datasets.
Additional file 4:
**Table for complete TRL results.** Classification performance of a TRL model on all datasets.
Additional file 5:
**Table for complete set of results for all algorithms.** Classification performance of all algorithms on all datasets.
Additional file 6:
**Table for complete set of results meta-analysis.** Classification performance of all non-transfer rule learning methods on all post meta-analysis datasets.
