# Supplementary material for: Knowledge transfer via classification rules using functional mapping for integrative modeling of gene expression data
Source: BMC Bioinformatics. 2015 Jul 23;16:226. doi: 10.1186/s12859-015-0643-8 (PMC4512094; doi:10.1186/s12859-015-0643-8)
Supplement: Additional file 1: — Supplementary methods. Additional information on discretization and semantic similarity. [file 12859_2015_643_MOESM1_ESM.docx]

# Methods

## Discretization with EBD

EBD has two components. First, it computes a Bayesian score to evaluate discretization, and second, it applies dynamic programming to search the space of likely discretizations efficiently. We briefly describe the method below:

Consider a given data set $D=\left[\left(A_{1},C_{1}\right), \left(A_{2},C_{2}\right), \ldots,\left(A_{k},C_{k}\right),\ldots,\left(A_{n},C_{n}\right)\right]$ of $n$ instance variables, where $D$ is sorted in increasing order of$A_{k}$, and $A_{k}$ and $C_{k}$ are, respectively, a real-value predictor variable of $A$ and the corresponding value of the target variable$C$. In addition, let $S$ represent a list of the first element of$C$. EBD defines a discretization model of the form$M\equiv\left(W,T,\text{Θ}\right)$, where $W$ represents the number of intervals in the discretization and $T$ is the discretization of$S$. Given a specific interval$i$, EBD models the distribution of the target variable $P(C|W=i)$ as a multinomial distribution with parameters$\left\{\theta_{i1}, \theta_{i2},\ldots,\theta_{ij},\ldots,\theta_{iJ}\right\}$, where the $J$s are indices to the distinct values$C$. Consequently, $\text{Θ}=\left\{\theta_{ij}\right\}$ represents all the multinomial distributions in$M$.

With an input data$D$, the EBD method calculates a Bayesian score for all likely discretizations of $S$ (i.e., all possible combinations of splitting $S$ into $i$ intervals). In the second phase of the method, EBD uses dynamic programming to search the space of possible discretizations to select the discretization that maximizes the Bayesian score.

***The Gene Ontology as information source***

The GO project [[19](#_ENREF_19)] maintains a dynamic, well structured, cross-species controlled vocabulary of terms that describe gene product characteristics and gene product annotation data [[20](#_ENREF_20)]. GO defines the relationships between terms with a directed acyclic graph, where each GO term is a node and an arc represents the relationship among the terms. The GO graph consists of three sub-ontologies covering three domains: cellular component, molecular function, and biological process. In this study, we focused on the biological process sub-ontology since gene products that form a functional module are likely to be involved in the same biological process or similar biological processes.

In the GO graph, several terms that are semantically similar annotate the same gene. To avoid redundancy, we clustered the semantically similar terms into functional themes [[17](#_ENREF_17)]. Below, we briefly describe the semantic similarity between two GO terms and how we quantified it.

***Semantic similarity***

Semantic similarity (SS) is a measure that tries to quantify the closeness between two GO terms based on the functional feature(s) that describe them. The intuition is that semantically similar GO terms are more likely to annotate genes found within the same FM. This means that a relatively high SS value among annotations of two genes suggests that they either can perform the same biological function or are found in the same biological process. Therefore, by using the SS measure, we were able to identify functionally related terms and cluster them accordingly. Several methods for measuring SS have been proposed, each having its strengths and drawbacks [[21](#_ENREF_21)]. We adopted the Wang et al [[22](#_ENREF_22)] method (see Additional file 1), because it takes advantage of the hierarchical structure of the GO to compute semantic similarity.

***Clustering semantically similar GO terms into functional themes***

Due to the complexity of the GO graph, several distinct terms can annotate the same genes with several degrees of specificity. Choosing a particular term to describe the underlying commonalities among a group of genes can be very challenging. However, most of these GO terms have a common underlying function, so grouping them into common functional themes is essential for reducing potential redundancies inherent in the GO annotations.

We implemented the spectral clustering algorithm [[23](#_ENREF_23)] to group GO terms according to the functional themes that underlie them. With inputs from a similarity graph, represented by a similarity matrix (), and the desired number of clusters, $k$, the spectral clustering algorithm tries to optimize the partitioning of $S$ into $k$ clusters such that objects in different clusters are dissimilar from each other while objects within the same cluster are similar to each other. For example, given a set of $n$ GO terms,$GO=\left\{go_{1},go_{2},\ldots,go_{n}\right\}$, we constructed a pairwise similarity matrix$S=\left[SS\left(go_{i},go_{j}\right)\right]$, where $SS\left(A,B\right)$ denotes the semantic similarity between GO terms $A$ and $B$ according to equation (3) above. Then, because identifying the optimal number of clusters (i.e. *k* value) is a challenge for most clustering algorithms, we searched for the *k* value that maximized the mean silhouette value [[24](#_ENREF_24)]. This value is a measure that determines the degree to which an object (i.e., a GO term) fits into the cluster (i.e., functional theme) to which it has been assigned. To assuage the discovery of spurious clusters, we discarded clusters with an average silhouette value of less than 0.5.

***Mapping of genes into functional modules***

In general, several functional themes map to the same gene and the converse is true. This mapping results into a many-to-many relationship between genes and functional themes. To group the genes into FMs as well as identify those that are potentially multifunctional, we mapped each gene to a functional theme containing at least one GO term that annotates it. Thus, the group of genes that map to a particular functional theme forms a functional module. Observe that the functional modules formed in this process are not disjointed.

## *Wang’s method of measuring semantic similarity*

Wang et al [[29](#_ENREF_29)] averred that in measuring the SS among GO terms, the locations of the common ancestors in relation to any specific term in the GO graph must be taken into consideration. In their proposed method, the semantics of a GO term is an aggregate contribution of all its ancestors. Therefore, for any GO term ***A***, the terms closer to it (e.g., parents) contribute more to its semantics than the terms farther in the graph over ***A*** (e.g., root). They computed SS as follows:

Define the directed acyclic graph (DAG) over term *A,* in the GO, as$DAG_{A}=\left(A,T_{A},E_{A}\right)$, where $T_{A}$ is the set of terms in$DAG_{A}$ (i.e., term ***A*** and all its ancestors), while $E_{A}$ is the set of edges in$DAG_{A}$. The S-value of *t* (where,$t\in DAG_{A}$) in relation to ***A*** measures the contribution to the semantics of the latter by the former. We denote the S-value of *t* in relation to ***A*** as $S_{A}\left(t\right)$ and define it formally as follows:

$\left\{\begin{matrix} \begin{matrix} S_{A}\left(A\right)=1 \\ \end{matrix} \\ S_{A}\left(t\right)=max\left\{w_{e}*S_{A}\left(t'\right)|t'\in childrenof\left(t\right)\right\} if t\neq A \end{matrix}\right.$ (1)

where $w_{e}$ is the semantic contribution factor for edge $e\in E_{A}$, linking term *t* with its child term *t’*. The contribution of term ***A*** to its own semantics is one. After obtaining the S-values for all terms *t* related to *A*, the semantic value of GO term ***A***,$SV\left(A\right)$, is computed as:

$SV\left(A\right)=\sum_{t\in T_{A}} S_{A}\left(t\right)$ (2)

Now, given two GO terms ***A*** and ***B***, the measure of the SS between them,$SS\left(A,B\right)$, is given by:

$SS\left(A,B\right)=\sum_{t\in T_{A}\cap T_{B}} \frac{S_{A}\left(t\right)+S_{B}\left(t\right)}{SV\left(A\right)+SV\left(B\right)}$ (3)

where $S_{A}\left(t\right)$ is the S-value of GO term *t* related to term ***A*** and $S_{B}\left(t\right)$ is the S-value of GO term *t* related to term ***B***. Using the SS as a basis, we constructed a similarity matrix, which served as input to the clustering algorithm.
