# Supplementary material for: Knowledge transfer via classification rules using functional mapping for integrative modeling of gene expression data
Source: BMC Bioinformatics. 2015 Jul 23;16:226. doi: 10.1186/s12859-015-0643-8 (PMC4512094; doi:10.1186/s12859-015-0643-8)
Supplement: Additional file 2: — Table of Functional modules. A list of functional modules that facilitated knowledge transfer for classification rule modeling on respective target datasets. [file 12859_2015_643_MOESM2_ESM.docx]

**Functional modules for learning to target datasets**

**IPF datasets**

**Emblom**

| Clusters | GO Functional Theme | Markers |
| --- | --- | --- |
| **FM1** | Regulation of kinase activity | *CBS, FCER1A, THY1* |
| **FM2** | Notch signaling | *BAI2, CNTNAP2, HEY1, IL13RA2, PKIG,* |
| **FM3** | Cardiac epithelial to mesenchymal transition | *HEY1* |
| **FM4** | Cell adhesion | *CDH2, CNTNAP2, THY1* |
| **FM5** | T cell receptor signaling pathway | *ASPN, CDH2, FCER1A, HEY1, THY1* |
| **FM6** | Brain development | *BAI2, CBS, CNTNAP2* |
| **FM7** | Protein homooligomerization | *DPYSL3, MPP6* |
| **FM8** | Pyrimidine nucleobase catabolic process | *CBS, DPYSL3, THY1* |
| **FM9** | Transcription | *HEY1, HR, PKIG* |
| **FM10** | Transsulfuration | *CBS* |
| **FM11** | Muscle cell differentiation | *CDH2, HEY1, SRD5A1* |
| **FM12** | Sex determination | *CBS, CNTNAP2, SRD5A1* |

**KangB**

| Clusters | GO Functional Theme | Markers |
| --- | --- | --- |
| **FM1** | Regulation of protein import | *PKIG, THY1* |
| **FM2** | Neuron recognition | *CNTNAP2, THY1* |
| **FM3** | Regulation of muscle cell differentiation | *CDH2, HEY1* |
| **FM4** | Cell adhesion | *CDH2, CNTNAP2, THY1* |
| **FM5** | Regulation of notch signaling pathway | *ASPN, CDH2, HEY1* |
| **FM6** | Cardiac epithelial to mesenchymal transition | *HEY1* |
| **FM7** | Cytoskeleton organization | *H2AFY2, MMP11, THY1* |
| **FM8** | Transcription | *HEY1, PKIG* |
| **FM9** | Myocardium morphogenesis | *HEY1* |
| **FM10** | Muscle cell differentiation | *CDH2, HEY1, SRD5A1* |
| **FM11** | Sex determination | *SRD5A1* |
| **FM12** | Cellular protein localization | *CNTNAP2* |

**Konishi**

| Clusters | GO Functional Theme | Markers |
| --- | --- | --- |
| **FM1** | Regulation of interleukin-3 biosynthesis | *FCERIA* |
| **FM2** | Transsulfuration | *CBS* |
| **FM3** | Epidermis development | *ASPN, BAI2, KLK7* |
| **FM4** | Superoxide metabolic process | *CBS* |
| **FM5** | Notch signaling pathway | *BAI2, HEY1, IL13RA2, PKIG* |
| **FM6** | Cardiac epithelial to mesenchymal transition | *HEY1* |
| **FM7** | Protein homooligomerization | *DPYSL3, MPP6* |
| **FM8** | Transcription | *HEY1, PKIG, HR* |
| **FM9** | Myocardium morphogenesis | *HEY1* |
| **FM10** | Cysteine catabolic process | *CBS* |

**Larsson**

| Clusters | GO Functional Theme | Markers |
| --- | --- | --- |
| **FM1** | Regulation of notch signaling | *ASPN, CDH2, HEY1, THY1* |
| **FM2** | Transsulfuration | *CBS* |
| **FM3** | Cell junction assembly | *CDH2, MMP11, THY1* |
| **FM4** | Superoxide metabolic process | *CBS* |
| **FM5** | Notch signaling pathway | *BAI2, CNTNAP2, HEY1, IL13RA2,* |
| **FM6** | Cellular protein localization | *CNTNAP2* |
| **FM7** | Pyrimidine nucleobase catabolic process | *CBS, DPYSL3, THY1* |
| **FM8** | Transcription | *HEY1, HR* |
| **FM9** | Myocardium morphogenesis | *HEY1* |
| **FM10** | Cell adhesion | *CDH2, CNTNAP2, THY1* |

**Pardo**

| Clusters | GO Functional Theme | Markers |
| --- | --- | --- |
| **FM1** | Muscle cell differentiation | *CDH2, CNTNAP2, DPYSL3, SRD5A1, THY1* |
| **FM2** | Transsulfuration | *CBS* |
| **FM3** | Cell junction assembly | *CDH2, THY1* |
| **FM4** | Superoxide metabolic process | *CBS* |
| **FM5** | Sex determination | *SRD5A1* |
| **FM6** | Regulation of protein kinase activity | *CBS, FCER1A, THY1* |
| **FM7** | Pyrimidine nucleobase catabolic process | *CBS, DPYSL3, THY1* |
| **FM8** | Transcription | *PKIG, HR* |
| **FM9** | G-protein coupled receptor signaling | *BAI2, CNTNAP2, IL13RA2, PKIG* |
| **FM10** | Cell adhesion | *CDH2, CNTNAP2, THY1* |
| **FM11** | Protein homooligomerization | *DPYSL3, MPP6* |
| **FM12** | Brain development | *BAI2, CBS, CNTNAP2, KLK7* |
| **FM13** | Regulation of interleukin-3 biosynthesis | *FCERIA* |

**Yang**

| Clusters | GO Functional Theme | Markers |
| --- | --- | --- |
| **FM1** | Notch signaling pathway | *BAI2, CNTNAP2, HEY1, IL13RA2, PKIG* |
| **FM2** | Transsulfuration | *CBS* |
| **FM3** | Cell junction assembly | *CDH2, THY1, H2AFY2, MMP11* |
| **FM4** | Protein cell localization | *CNTNAP2* |
| **FM5** | Sex determination | *SRD5A1* |
| **FM6** | Superoxide metabolic process | *CBS* |
| **FM7** | Transcription | *PKIG, HEY1, HR* |
| **FM8** | Cardiac epithelial to mesenchymal transition | *HEY1* |
| **FM9** | Cell adhesion | *CDH2, CNTNAP2, THY1* |
| **FM10** | Myocardium morphogenesis | *HEY1* |
| **FM11** | Brain development | *BAI2, CNTNAP2* |
| **FM12** | T cell receptor signaling | *CDH2, FCER1A, THY1* |

**Brain cancer datasets**

**Gravendeel**

| Clusters | GO Functional Theme | Markers |
| --- | --- | --- |
| **FM1** | DNA repair | *MRE11A, SMC4, USP47* |
| **FM2** | Regulation of kinase activity | *AP2B1, CDC37, DVL1, PPP2R5A* |
| **FM3** | Protein folding | *ARAF, B3GAT1, CASP5, CSNK1G1, ENPEP, FBXO21, JAG1, NPEPPS, P4HB, PJA2, USP47, ZMPSTE24* |
| **FM4** | Organ development | *ALDH5A1, ASPN, COL5A2, DAZL, DVL1, ENPEP, FBN1, FKTN, GABRB3, IFRD1, JAG1, MGP, NDST1, NHLH2, PBX1, RPS6KA3, RYR1, ZMYM3* |
| **FM5** | Cell differentiation | *ACTG1, ALDH6A1, AP2B1, COL4A2, COL5A2, CRB1, DVL1, GABRB3, IFRD1, JAG1, MGP, MSR1, MYO10, NHLH2, PBX1, RPS6KA3, RYR1, USH1C, ZMYM3* |
| **FM6** | Apoptosis process | *ADAMTSL4, ARAF, CASP5, P2RX1, RPS6KA3, TEX261, TRIAP1, USP47* |
| **FM7** | Transport | *ADCY3, AP2B1, ATP9A, GABRB3, MSR1, MYO10, P2RX1, RYR1, ZFPL1* |
| **FM8** | Transcription | *COL4A2, DVL1, JAG1, MRE11A, NHLH2, NKRF, NR1D2, PBX1, RPS6KA3, STRAP, USP47, ZFPL1, ZMYND11* |
| **FM9** | Signal transduction | *ADCY3, AP2B1, ARAF, CD97, CSNK1G1, CXCL6, DVL1, GABRB3, JAG1, LANCL1, NDST1, NR1D2, P2RX1, PPP2R5A, RPS6KA3, STC1* |
| **FM10** | Toll-like receptor signaling | *RPS6KA3* |
| **FM11** | Metabolic processes | *ALDH5A1, ALDH6A1, B3GAT1, LDHA, NDST1, P4HB* |

**Freije**

| Clusters | GO Functional Theme | Markers |
| --- | --- | --- |
| **FM1** | DNA repair | *BLVRA, CSTF2T, DHX9, MCM2, MRE11A, SMC4* |
| **FM2** | Regulation of protein phosphorylation | *ADCY7, ADORA1, ARAF, MRE11A, PPP2R5A, VEGFA* |
| **FM3** | Cell cycle | *MCM2, MRE11A, PELO, SMC4, USH1C* |
| **FM4** | Organ development | *ADORA1, CDC37, DVL1, FKTN, MRE11A, PPP2R5A, VEGFA* |
| **FM5** | Cell differentiation | *ACTG1, AP2B1, COL4A2, COL5A2, CRB1, DVL1, EFNB2, GABRB3, IFRD1, JAG1, MYO10, NHLH2, RYR1, STRN, USH1C, VEGFA, ZIC3* |
| **FM6** | Transport | *ABCC10, ADCY3, ADCY7, ADORA1, AP2B1, ATP9A, CDC37, DVL1, GABRB3, LRMP, MSR1, MYO10, P2RX1, PDIA4, RYR1, VEGFA, ZFPL1* |
| **FM7** | Signal transduction | *ADCY3, ADCY7, ADORA1, AP2B1, ARAF, CD97, CSNK1G1, CXCL6, DVL1, EFNB2, GABRB3, IGFBP2, JAG1, LANCL1, NDST1, NR1D2, P2RX1, PPP2R5A, RPS6KA3, SGPL1, STC1, STRN, VEGFA* |
| **FM8** | Cardiac morphogenesis | *COL4A2, EFNB2, ENPEP, IGFBP2, JAG1, RYR1, SGPL1, VEGFA* |
| **FM9** | Response to glucose stimuli | *ADCY7, COL4A2, COL5A2, CTSL2, CYP2E1, IGFBP2, JAG1, NDST1, P2RX1, RYR1, STC1, VEGFA* |
| **FM10** | Metabolic processes | *ADCY7, ADORA1, ALDH5A1, ALDH6AI, BLVRA, B3GATI, CYP2E1, LDHA, NDST1, P2RX1, P4HB, PBX1, PDIA4, SGPL1* |
| **FM11** | Regulation of angiogenesis | *COL4A2, EFNB2, VEGFA* |
| **FM12** | Response to stress | *ADORA1, CASP5, CD97, CTSL2, CXCL6, CYO2E1, DHX9, IGFBP2, KLRC3, LDHA, MRE11A, MSR1, NDST1, NPEPPS, RYR1, STC1, TRIAP1, USP47, VEGFA* |

**Phillips**

| Clusters | GO Functional Theme | Markers |
| --- | --- | --- |
| **FM1** | Apoptotic processes | *ADAMTSL4, ADORA1, ARAF, CASP5, CTSL2, P2RX1, RPS6KA3, SGPL1, TEX261, TRIAP1, USP47, VEGFA* |
| **FM2** | Regulation of protein phosphorylation | *ADCY3, ADCY7, ADORA1, ARAF, CDC37, MRE11A, PPP2R5A, VEGFA* |
| **FM3** | Cell differentiation | *ACTG1, ALDH6A1, AP2B1, COL4A2, COL5A2, CRB1, EFNB2, GABRB3, MGP, MSR1, MYO10, NHLH2, PBX1, RPS6KA3, SGPL1, STRN, USH1C, VEGFA, ZIC3* |
| **FM4** | Transport | *ABCC10, ADCY3, ADCY7, ADORA1, AP2B1, ATP9A, CDC37, GABRB3, LRMP, MSR1, MYO10, P2RX1, PDIA4, VEGFA, ZFPL1* |
| **FM5** | Signal transduction | *ADCY3, ADCY7, ADORA1, AP2B1, ARAF, CD97, CRB1, CSNK1G1, CXCL6, EFNB2, GABRB3, IGFBP2, NR1D2, P2RX1, PPP2R5A, RPS6KA3, SGPL1, STC1, STRN, VEGFA* |
| **FM6** | Cell cycle | *MCM2, MRE11A, RPS6KA3, SMC4, USH1C, USP47, ZMYND11* |
| **FM7** | Response to glucose stimuli | *ADCY3, ADCY7, COL4A2, COL5A2, CTSL2, CYP2E1, IGFBP2, P2RX1, RPS6KA3, STC1, STRAP, VEGFA* |
| **FM8** | Toll-like receptor signaling | *RPS6KA3* |
| **FM9** | Transcription | *ADCY7, BTAF1, COL4A2, DAZL, FBN1, FKTN, MRE11A, NHLH2, NKRF, NR1D2, PBX1, RPS6KA3, STRAP, USP47, VEGFA, ZFPL1, ZIC3, ZMYND11, ZNF187* |
| **FM10** | Lipid metabolism | *ADORA1, ALDH5A1, CYP2E1, P2RX1, P4HB, PBX1, PPP2R5A, SGPL1* |
| **FM11** | Cardiac morphogenesis | *ASPN, COL4A2, COL5A2, CTSL2, EFNB2, FBN1, FKTN, GABRB3, IGFBP2, MCAM, MGP, PBX1, SGPL1, USH1C, VEGFA, ZIC3* |

**Paugh**

| Clusters | GO Functional Theme | Markers |
| --- | --- | --- |
| **FM1** | Cardiac morphogenesis | *ADORA1, ALDH5A1, ASPN, COL4A2, COL5A2, CTSL2, DVL1, EFNB2, ENPEP, FBN1, FKTN, GABRB3, IFRD1, IGFBP2, JAG1, MCAM, MGP, NDST1, PBX1, PCDH8, RPS6KA3, RYR1, SGPL1, USH1C, VEGFA* |
| **FM2** | Apoptotic processes | *ADAMTSL4, ADORA1, CASP5, FKTN, MCM2, P2RX1, RPS6KA3, SGPL1, STRN, TEX261, TRIAP1, USP47, VEGFA* |
| **FM3** | Cell differentiation | *ALDH6A1, AP2B1, COL4A2, COL5A2, CRB1, CTSL2, DVL1, EFNB2, GABRB3, IFRD1, JAG1, MGP, MYO10, PBX1, RPS6KA3, RYR1, SGPL1, STRN, USH1C, VEGFA, ZIC3* |
| **FM4** | Transport | *ABCC10, ADCY3, ADCY7, ADORA1, AP2B1, ATP9A, GABRB3, MSR1, MYO10, P2RX1, RYR1, ZFPL1* |
| **FM5** | Regulation of signal transduction | *AP2B1, ASPN, CDC37, DVL1, FBN1, FKTN, IGFBP2, JAG1, PJA2, STRAP, VEGFA, ZMYND11* |
| **FM6** | DNA repair | *ALDH6A1, BLVRA, COL4A2, CSTF2T, CYP2E1, DHX9, DNASE1L1, DVL1, MCM2, NKRF, NR1D2, PBX1, SMC4, STRAP, USP47, ZIC3, ZMYND11, ZNF187* |
| **FM7** | Cell-cell signaling | *ADCY3, ADCY7, ADORA1, ALDH5A1, AP2B1, CD97, CRB1, CXCL6, DVL1, EFNB2, ENPEP, GABRB3, P2RX1, PCDH8, RPS6KA3,* |
| **FM8** | Toll-like receptor signaling | *RPS6KA3* |
| **FM9** | Cell-cycle | *CD97, CRB1, CTSL2, EFNB2, ENPEP, MCAM, MCM2, PCDH8, PELO, PPFIBP1, RPS6KA3, SMC4, TPM3, USH1C, VEGFA, ZMYND11* |
| **FM10** | Chromosome condensation | *MCM2, SMC4* |

**Sun**

| Clusters | GO Functional Theme | Markers |
| --- | --- | --- |
| **FM1** | DNA repair | *ALDH6A1, CSTF2T, DHX9, DNASE1L1, MCM2, MRE11A, SMC4, STRAP, USP47* |
| **FM2** | Apoptotic processes | *ADAMTSL4, ADORA1, ARAF, CASP5, CTSL2, FKTN, MCM2, MRE11A, P2RX1, RPS6KA3, SGPL1, STRN, TEX261, TRIAP1, USP47, VEGFA* |
| **FM3** | Organ development | *ADORA1, ALDH5A1, ASPN, COL5A2, CTSL2, DVL1, EFNB2, ENPEP, GABRB3, JAG1, MGP, NDST1, NHLH2, RPS6KA3, RYR1, SGPL1, USH1C, VEGFA, ZIC3* |
| **FM4** | Cell differentiation | *ALDH6A1, EFNB2, IFRD1, JAG1, MGP, NHLH2, SGPL1, VEGFA, ZIC3, MCAM, MGP, PBX1, SGPL1, USH1C, VEGFA, ZIC3* |
| **FM5** | Transport | *ABCC10, ADCY3, ADCY7, ADORA1, ATP9A, GABRB3, LRMP, MSR1, P2RX1, PDIA4, RYR1, VEGFA, ZFPL1* |
| **FM6** | Signal transduction | *ADCY3, ADCY7, ADORA1, ARAF, CD97, CSNK1G1, DVL1, EFNB2, GABRB3, IGFBP2, JAG1, LANCL1, NDST1, NR1D2, P2RX1, RPS6KA3, SGPL1, STC1, STRN, VEGFA* |
| **FM7** | Cell proliferation | *ADORA1, ENPEP, IGFBP2, JAG1, MRE11A, MSR1, PELO, RPS6KA3, STRN, TEX261, USP47, VEGFA, ZMYND11, RPS6KA3, SMC4, STC1, TPM3, TRIAP1, USP47, VEGFA* |
| **FM8** | Lipid metabolism | *ADORA1, ALDH5A1, CYP2E1, P2RX1, P4HB, SGPL1, RPS6KA3, STRN, USP47, VEGFA, ZMYND11* |
| **FM9** | Response to glucose stimuli | *ADCY3, ADCY7, COL4A2, COL5A2, CTSL2, CYP2E1, IGFBP2, JAG1, NDST1, P2RX1, RPS6KA3, RYR1, STC1, STRAP, VEGFA* |
| **FM10** | Transcription | *ADCY7, BTAF1, COL4A2, DAZL, DVL1, JAG1, MRE11A, NHLH2, NR1D2, RPS6KA3, STRAP, USP47, VEGFA, ZFPL1, ZIC3, ZMYND11, ZNF187, RPS6KA3, SERPINH1, SGPL1, STC1, TPM3, USH1C, VEGFA, ZIC3, ZMYM3* |
| **FM11** | Response to stress | *ADORA1, CALCOCO2, CD97, CYP2E1, DVL1, IGFBP2, KLRC3, MSR1, NDST1, RPS6KA3, RYR1, SERPINH1, STC1, STRN, USP47, VEGFA* |

**Yamanaka**

| Clusters | GO Functional Theme | Markers |
| --- | --- | --- |
| **FM1** | Activation of kinase activity | *ADCY3, ADCY7, ADORA1, ARAF, CDC37, DVL1, MRE11A, PPP2R5A, VEGFA* |
| **FM2** | Apoptotic processes | *ADAMTSL4, ADORA1, ARAF, CASP5, CTSL2, FKTN, MCM2, MRE11A, P2RX1, RPS6KA3, SGPL1, STRN, TEX261, TRIAP1, USP47, VEGFA* |
| **FM3** | Organ development | *ADORA1, ALDH5A1, COL5A2, CTSL2, DVL1, EFNB2, ENPEP, FBN1, FKTN, GABRB3, IFRD1, IGFBP2, JAG1, MGP, NDST1, NHLH2, PBX1, RPS6KA3, RYR1, SGPL1, VEGFA, ZIC3* |
| **FM4** | Transport | *ABCC10, ADCY3, ADCY7, ADORA1, AP2B1, ATP9A, CDC37, GABRB3, LRMP, MSR1, MYO10, PDIA4, RYR1, VEGFA, ZFPL1* |
| **FM5** | Signal transduction | *ADCY3, ADCY7, ADORA1, AP2B1, ARAF, CD97, CXCL6, DVL1, EFNB2, GABRB3, IGFBP2, JAG1, LANCL1, NDST1, NR1D2, PPP2R5A, RPS6KA3, SGPL1, STC1, STRN, VEGFA* |
| **FM6** | Cell proliferation | *ADAMTSL4, EFNB2, IGFBP2, JAG1, MSR1, MYO10, PBX1, RPS6KA3, TEX261, USP47, VEGFA* |
| **FM7** | Lipid metabolism | *ADORA1, ALDH5A1, BLVRA, CYP2E1, P4HB, PBX1, PPP2R5A, SGPL1* |
| **FM8** | Response to glucose stimuli | *ADCY3, ADCY7, AP2B1, COL4A2, COL5A2, CTSL2, CYP2E1, IGFBP2, JAG1, NDST1, RPS6KA3, RYR1, STC1, STRAP, VEGFA* |
| **FM9** | DNA repair | *MRE11A, SMC4, USP47* |
| **FM10** | Response to stress | *ADORA1, CASP5, CD97, CTSL2, CXCL6, DHX9, IGFBP2, KLRC3, LDHA, MRE11A, NDST1, NPEPPS, RPS6KA3, RYR1, STC1, TRIAP1, USP47, VEGFA* |

**Prostate cancer datasets**

**Nanni**

| Clusters | GO Functional Theme | Markers |
| --- | --- | --- |
| **FM1** | Cardiac and urinary organ morphogenesis | *ACTL6A, ANXA2, COQ7, ERG, FOSB, FZD7, GATA3, GATM, GJA1, LEPR, MYL6, NELL1, NFATC3, RCAN2, SNAI2, SOX9, WHSC1, WIF1* |
| **FM2** | Lipid metabolism | *ABCA2, ADCY2, AMACR, C3, COQ7, GATA3, GATM, LEPR, MTMR7, NFATC3, PARG, SNAI2* |
| **FM3** | Regulation of chemokine production | *C3, DARC, GATA3, SCGB1A1, SNAI2, SOX9* |
| **FM4** | Signal transduction | *ACTL6A, ADCY2, C3, DARC, ERG, FZD7, GATA3, GDI1, GJA1, LEPR, MAP3K14, PRKCB, RCAN2, SCGB1A1, SNAI2, SOX9, WIF1* |
| **FM5** | Chemotaxis | *ABCA2, ACTA2, GATA3, GATM, GDI1, GJA1, JUND, PARG, SCGB1A1* |
| **FM6** | Transcription | *ACTL6A, ERG, GATA3, JUND, NFATC3, NFYB, POLR2H, PRKCB, TCEAL4, WHSC1* |
| **FM7** | Regulation of transcription | *ABCA2, ACTL6A, FOSB, FZD7, GATA3, JUND, NFATC3, NFYB, POLR2H, PRKCB, SCGB1A1, SNAI2, SOX9, TCEAL4, WHSC1* |
| **FM8** | Translation | *ACTL6A, DNAJC11, EEF2, ERG, MTMR7, POLR2H, PRKCB, RPS29, SEC23A, USP33* |
| **FM9** | Cellular response to cytokines | *ADCY2, ANXA2, DARC, FOSB, FZD7, GATA3, JUND, NFATC3, PRKCB, SNAI2, SOX9* |
| **FM10** | Cell proliferation | *ERG, FZD7, GATA3, GJA1, MARCKSL1, NELL1, SNAI2, SOX9* |
| **FM11** | Transport | *ABCA2, ADCY2, C3, DYNLT1, FZD7, GATA3, GDI1, GJA1, KCNN4, PRKCB, REPS1, RPS29, SEC23A, SLC43A1, SNAI2, SNX7, USP33* |
| **FM12** | Cell differentiation | *ACTA2, COQ7, DYNLT1, FZD7, GATA3, JUND, MEA1, NELL1, NFATC3, SNAI2, SOX9, WIF1* |

**Singh**

| Clusters | GO Functional Theme | Markers |
| --- | --- | --- |
| **FM1** | Regulation of cell proliferation | *FZD7, GJA1, MARCKSL1, NELL1, SNAI2* |
| **FM2** | Regulation of osteoblast differentiation | *SNAI2, USP33* |
| **FM3** | Signal transduction | *ADCY2, BCAM, CCL1, DARC, DYNLT1, FZD7, GDI1, GJA1, LEPR, MAP3K14, RCAN2, SNAI2, USP33* |
| **FM4** | Chemotaxis | *ABCA2, CCL1, GATA3, GATM, GDI1, JUND, SCGB1A1* |
| **FM5** | Transcription | *ABCA2, FOSB, FZD7, JUND, MUC1, NFATC3, RPS29, SNAI2, TCEAL4, WHSC1* |
| **FM6** | Translation | *EEF2, MUC1, RPS29, SEC23A* |
| **FM7** | Transport | *ABCA2, ADCY2, ATOX1, GJA1, REPS1, SEC23A, USP33* |
| **FM8** | Protein folding | *DNAJC11, EEF2, MTMR7, MUC1, RPS29, SEC23A, USP33* |
| **FM9** | mRNA processing | *BCAS2, RPS29* |

**Varambally**

| Clusters | GO Functional Theme | Markers |
| --- | --- | --- |
| **FM1** | Cardiac and urinary organ morphogenesis | *ACTL6A, COQ7, FZD7, GATA3, GATM, GJA1, NFATC3, SNAI2, SOX9, WHSC1* |
| **FM2** | Lipid metabolism | *ABCA2, ADCY2, AMACR, C3, COQ7, GATA3, GATM, LEPR, MTMR7, NFATC3, PARG, SNAI2* |
| **FM3** | Regulation of chemokine production | *C3, DARC, GATA3, SCGB1A1, SNAI2, SOX9* |
| **FM4** | Signal transduction | *ACTL6A, ADCY2, BCAM, C3, CCL1, DARC, ERG, FZD7, GATA3, GDI1, GJA1, LEPR, MAP3K14, MUC1, PRKCB, RCAN2, SCGB1A1, SNAI2, SOX9, WIF1* |
| **FM5** | Transcription | *ACTL6A, BCAS2, ERG, GATA3, JUND, NFATC3, NFYB, POLR2H, PRKCB, RPS29, TCEAL4, WHSC1* |
| **FM6** | Regulation of transcription | *ABCA2, ACTL6A, FOSB, FZD7, GATA3, JUND, MUC1, NFATC3, NFYB, POLR2H, PRKCB, SCGB1A1, SNAI2, SOX9, TCEAL4, WHSC1* |
| **FM7** | Translation | *DNAJC11, ERG, MTMR7, MUC1, POLR2H, PRKCB, RPS29, SEC23A* |
| **FM8** | Cellular response to cytokines | *ABCA2, ADCY2, ANXA2, DARC, FOSB, FZD7, GATA3, GATM, GJA1, JUND, NFATC3, PRKCB, SCGB1A1, SNAI2, SOX9* |
| **FM9** | Regulation of cell proliferation | *FZD7, GATA3, GJA1, MARCKSL1, NELL1, SNAI2, SOX9* |
| **FM10** | Transport | *ABCA2, ADCY2, ATOX1, C3, GJA1, KCNN4, PRKCB, REPS1, SEC23A, SLC43A1* |
| **FM11** | Regulation of cell differentiation | *DYNLT1, FZD7, GATA3, JUND, NELL1, SNAI2, SOX9, WIF1* |
| **FM12** | Regulation of signal transduction | *C3, DYNLT1, FZD7, GATA3, GDI1, GJA1, KCNN4, MAP3K14, PRKCB, SNAI2, SOX9* |
| **FM13** | Histone acetylation and methylation | *ACTL6A, ADCY2, C3, FZD7, GATA3, GJA1, MUC1, NELL1, PRKCB, SNAI2, SOX9* |
| **FM14** | Protein transport | *ABCA2, DYNLT1, FZD7, GATA3, GDI1, KCNN4, PRKCB, RPS29, SEC23A, SNAI2, SNX7* |

**Wallace**

| Clusters | GO Functional Theme | Markers |
| --- | --- | --- |
| **FM1** | Lipid metabolism | *ADCY2, AMACR, C3, COQ7, FZD7, GATA3, GATM, MTMR7, PARG, SNAI2* |
| **FM2** | Regulation of chemokine production | *C3, DARC, GATA3, SCGB1A1, SNAI2, SOX9* |
| **FM3** | Signal transduction | *ACTL6A, ADCY2, C3, CCL1, DARC, ERG, FZD7, GATA3, GJA1, MAP3K14, PRKCB, RCAN2, SCGB1A1, SNAI2, SOX9, WIF1* |
| **FM4** | Chemotaxis | *ACTA2, ACTL6A, ADCY2, ANXA2, C3, CCL1, EEF2, FOSB, GATA3, GATM, GJA1, JUND, PARG, POLR2H, RPS29, SCGB1A1, SEC23A, SNAI2* |
| **FM5** | Transcription | *ACTL6A, ERG, GATA3, JUND, NFYB, POLR2H, PRKCB, TCEAL4* |
| **FM6** | Regulation of transcription | *ACTL6A, FOSB, FZD7, GATA3, JUND, MUC1, NFYB, POLR2H, PRKCB, SCGB1A1, SNAI2, SOX9, TCEAL4* |
| **FM7** | Translation | *ACTL6A, ADCY2, C3, EEF2, ERG, GATA3, GJA1, MTMR7, MUC1, POLR2H, PRKCB, RPS29, SEC23A, SNAI2, SOX9, USP33* |
| **FM8** | Cellular response to cytokines | *ADCY2, ANXA2, DARC, FOSB, FZD7, GATA3, JUND, PRKCB, SNAI2, SOX9* |
| **FM9** | Cell proliferation | *ERG, FZD7, GATA3, GJA1, MARCKSL1, SNAI2, SOX9* |
| **FM10** | Transport | *ADCY2, ATOX1, C3, GJA1, KCNN4, PRKCB, SEC23A, SLC43A1, USP33* |
| **FM11** | Regulation of signal transduction | *C3, FZD7, GATA3, GJA1, KCNN4, MAP3K14, PRKCB, SNAI2, SOX9, USP33* |
| **FM12** | Apoptotic process | *GATA3, GJA1, MUC1, PRKCB, SNAI2, SOX9* |

**Yu et al**

| Clusters | GO Functional Theme | Markers |
| --- | --- | --- |
| **FM1** | Cardiac and urinary organ morphogenesis | *ACTL6A, FZD7, GATA3, GJA1, MEA1, MYL6, NFATC3, SOX9* |
| **FM2** | Lipid metabolism | *ABCA2, ADCY2, AMACR, C3, COQ7, GATA3, GATM, LEPR, MTMR7, NFATC3, PARG, SNAI2* |
| **FM3** | Regulation of chemokine production | *C3, GATA3, SCGB1A1, SNAI2, SOX9* |
| **FM4** | Signal transduction | *ACTL6A, ADCY2, BCAM, C3, CCL1, ERG, FZD7, GATA3, GDI1, GJA1, LEPR, MAP3K14, MUC1, PRKCB, RCAN2, SCGB1A1, SNAI2, SOX9, WIF1* |
| **FM5** | Transcription | *ACTL6A, ERG, GATA3, NFATC3, NFYB, POLR2H, PRKCB, RPS29, TCEAL4, WHSC1* |
| **FM6** | Regulation of transcription | *ABCA2, ACTL6A, FZD7, GATA3, MUC1, NFATC3, NFYB, POLR2H, PRKCB, SCGB1A1, SNAI2, SOX9, TCEAL4, WHSC1* |
| **FM7** | Cellular response to cytokines | *ABCA2, ADCY2, FZD7, GATA3, GATM, GJA1, JUND, NFATC3, PRKCB, SCGB1A1, SNAI2, SOX9* |
| **FM8** | Regulation of cell proliferation | *ERG, FZD7, GATA3, GJA1, NELL1, SNAI2, SOX9* |
| **FM9** | Transport (ion) | *ABCA2, ADCY2, ATOX1, C3, GJA1, KCNN4, PRKCB, REPS1, SEC23A, SLC43A1, USP33* |
| **FM10** | Regulation of signal transduction | *C3, DYNLT1, FZD7, GATA3, GDI1, GJA1, KCNN4, MAP3K14, PRKCB, SNAI2, SOX9, USP33* |
| **FM11** | Protein post-translational modification | *DNAJC11, EEF2, ERG, MTMR7, MUC1, POLR2H, PRKCB, SEC23A, USP33* |
| **FM12** | Intracellular protein transport | *ABCA2, DYNLT1, FZD7, GATA3, GDI1, KCNN4, PRKCB, SEC23A, SNAI2, SNX7* |
| **FM13** | mRNA processing | *ACTL6A, BCAS2, POLR2H* |

**Welsh et al**

| Clusters | GO Functional Theme | Markers |
| --- | --- | --- |
| **FM1** | Cardiac and urinary organ morphogenesis | *ACTL6A, ANXA2, COQ7, GATA3, GATM, GJA1, MEA1, MYL6, NELL1, NFATC3, SNAI2, SOX9, WHSC1* |
| **FM2** | Lipid metabolism | *ABCA2, ADCY2, AMACR, C3, COQ7, GATA3, GATM, LEPR, MTMR7, NFATC3, PARG, SNAI2* |
| **FM3** | Regulation of chemokine production | *C3, DARC, GATA3, SCGB1A1, SNAI2, SOX9* |
| **FM4** | Signal transduction | *ACTL6A, ADCY2, BCAM, C3, CCL1, DARC, ERG, GATA3, GDI1, GJA1, LEPR, MAP3K14, MUC1, PRKCB, SCGB1A1, SNAI2, SOX9, WIF1* |
| **FM5** | Transcription | *ACTL6A, EEF2, ERG, GATA3, JUND, NFATC3, NFYB, POLR2H, PRKCB, RPS29, TCEAL4, WHSC1* |
| **FM6** | Negative regulation of transcription | *FOSB, GATA3, JUND, MUC1, SNAI2, SOX9, WHSC1* |
| **FM7** | Chemotaxis | *ABCA2, ACTA2, ATOX1, C3, CCL1, COQ7, DARC, FOSB, GATA3, GATM, GDI1, GJA1, JUND, KCNN4, MAP3K14, MUC1, NFATC3, PARG, SCGB1A1, SNAI2, SOX9* |
| **FM8** | Regulation of cell proliferation | *ERG, GATA3, GJA1, MARCKSL1, NELL1, SNAI2, SOX9* |
| **FM9** | Transport | *ABCA2, ADCY2, ATOX1, C3, GJA1, KCNN4, PRKCB, REPS1, SEC23A, SLC43A1, USP33* |
| **FM10** | Apoptotic process | *GATA3, GJA1, MUC1, PRKCB, SNAI2, SOX9* |
| **FM11** | Regulation of signal transduction | *C3, DYNLT1, GATA3, GDI1, GJA1, KCNN4, MAP3K14, PRKCB, SNAI2, SOX9, USP33* |
| **FM12** | Cellular response to cytokine stimuli | *ABCA2, ADCY2, ANXA2, DARC, FOSB, GATA3, GATM, GJA1, JUND, NFATC3, PRKCB, SCGB1A1, SNAI2, SOX9* |
| **FM13** | Intracellular protein transport | *ABCA2, DYNLT1, GATA3, GDI1, KCNN4, PRKCB, RPS29, SEC23A, SNAI2, SNX7* |
| **FM14** | Post-translational protein modification | *ACTL6A, ADCY2, C3, DNAJC11, EEF2, ERG, GATA3, GJA1, MTMR7, MUC1, NELL1, POLR2H, PRKCB, RPS29, SEC23A, SNAI2, SOX9, USP33* |
| **FM15** | mRNA processing | *ACTL6A, BCAS2, GATM, POLR2H, RPS29* |
