# Supplementary material for: Knowledge transfer via classification rules using functional mapping for integrative modeling of gene expression data
Source: BMC Bioinformatics. 2015 Jul 23;16:226. doi: 10.1186/s12859-015-0643-8 (PMC4512094; doi:10.1186/s12859-015-0643-8)
Supplement: Additional file 4: — Table for complete TRL results. Classification performance of a TRL model on all datasets. [file 12859_2015_643_MOESM4_ESM.pdf]

	TRL							
**Target**	**Source**							**Baseline**
Brain								
	Freije	Gravendeel	Paugh	Petalidis	Phillips	Sun	Yamanaka	
Freije		0.75(0.04)	**0.77(0.05)**	0.72(0.05)	**0.78(0.05)**	0.76(0.05)	0.76(0.04)	0.76(0.04)
Gravendeel	0.49(0.00)		0.49(0.00)	0.49(0.00)	0.49(0.00)	0.49(0.00)	0.49(0)	0.49(0.00)
Paugh	0.51(0.07)	0.50(0.08)		0.51(0.07)	**0.5(0.08)**	**0.52(0.06)**	0.51(0.07)	0.51(0.07)
Petalidis	0.82(0.05)	**0.88(0.07)**	0.81(0.07)		0.78(0.06)	**0.85(0.05)**	0.81(0.07)	0.83(0.06)
Phillips	**0.69(0.04)**	**0.73(0.07)**	**0.67(0.05)**	**0.68(0.04)**		**0.67(0.05)**	**0.67(0.06)**	0.66(0.05)
Sun	0.73(0.06)	0.7(0.06)	0.73(0.06)	0.73(0.06)	0.73(0.06)		0.72(0.07)	0.73(0.06)
Yamanaka	0.43(0.05)	0.50(0.09)	0.50(0.09)	0.50(0.09)	0.50(0.09)	0.50(0.09)		0.50(0.09)
IPF								
	Emblom	KangA	KangB	Konishi	Larsson	Pardo	Yang	
Emblom		0.97(0.02)	0.97(0.02)	0.94(0.03)	0.97(0.02)	0.97(0.02)	0.97(0.02)	0.97(0.02)
KangA	0.86(0.07)		0.86(0.07)	**0.93(0.05)**	0.86(0.07)	0.86(0.07)	0.86(0.07)	0.86(0.07)
KangB	0.83(0.05)	**0.91(0.04)**		**0.88(0.04)**	**0.85(0.06)**	0.83(0.05)	0.83(0.05)	0.83(0.05)
Konishi	0.78(0.07)	**0.81(0.05)**	**0.83(0.05)**		0.78(0.07)	0.78(0.07)	0.78(0.07)	0.78(0.07)
Larsson	0.75(0.25)	0.75(0.25)	0.75(0.25)	0.75(0.25)		0.75(0.25)	0.75(0.25)	0.75(0.25)
Pardo	0.85(0.11)	0.82(0.07)	0.80(0.11)	**0.90(0.07)**	0.85(0.11)		0.85(0.11)	0.85(0.11)
Yang	0.94(0.06)	0.89(0.07)	0.89(0.07)	0.94(0.06)	0.94(0.06)	0.94(0.06)		0.94(0.06)
Prostate								
	Lapointe	Nanni	Singh	Varambally	Wallace	Welsh	Yu	
Lapointe		0.93(0.03)	0.89(0.05)	0.9(0.03)	0.93(0.03)	0.91(0.04)	0.91(0.04)	0.93(0.03)
Nanni	0.54(0.14)		0.54(0.14)	0.54(0.14)	0.46(0.11)	0.54(0.14)	0.54(0.14)	0.54(0.14)
Singh	0.88(0.03)	0.89(0.03)		0.88(0.03)	0.89(0.03)	0.89(0.03)	0.89(0.03)	0.89(0.03)
Varambally	0.83(0.17)	0.83(0.17)	0.83(0.17)		**1.00(0.00)**	**1.00(0.00)**	**1.00(0.00)**	0.83(0.17)
Wallace	**0.81(0.06)**	**0.78(0.07)**	0.73(0.06)	0.76(0.07)		0.76(0.07)	**0.79(0.06)**	0.76(0.07)
Welsh	0.92(0.06)	**0.94(0.06)**	0.85(0.08)	0.89(0.07)	0.89(0.07)		**0.95(0.03)**	0.92(0.06)
Yu	**0.90(0.04)**	**0.90(0.06)**	0.87(0.06)	0.88(0.06)	0.88(0.06)	0.88(0.06)		0.88(0.06)
